# Anatomical and physiological trade-offs underlying the leaf economics spectrum in tropical Andean montane forests

**DOI:** 10.1093/aob/mcag131

**Published:** 2026-05-14

**Authors:** Dennis Castillo-Figueroa, Juan M Posada

**Affiliations:** School of Sciences and Engineering, Universidad del Rosario, Kr 26 # 63B-48, Bogotá 111311, Colombia; School of Sciences and Engineering, Universidad del Rosario, Kr 26 # 63B-48, Bogotá 111311, Colombia

**Keywords:** Functional traits, ecophysiology, photosynthesis, leaf anatomy, Andean forests, ecological strategies, tropical montane forests

## Abstract

**Background and aims:**

The leaf economic spectrum (LES) offers a comprehensive framework that elucidates the global covariation of leaf functional traits based on an investment and return analysis in leaf tissues, and reflects resource-acquisitive and resource-conservative plant strategies. Yet, this framework has focused on whole-leaf morphological, chemical and physiological traits, offering little insight about the role of the underlying anatomy. Anatomical traits vary widely and are likely to play a pivotal role in determining whole-organ traits, and also influence community assembly and ecosystem function. Here, using leaf anatomical metrics in 63 tropical Andean plant species we aimed to understand the fundamental anatomical trade-offs underlying the LES in these mountain forests.

**Methods:**

We measured 50 traits (41 anatomical and 9 foliar) and used Spearman correlations, principal component analysis and reduced major axis regressions to examine the physiological trade-offs between leaf anatomical characteristics and foliar traits associated with the LES.

**Key Results:**

Thicker leaf tissues and larger vascular bundles were linked to conservative strategies, whereas traits related to carbon fixation – particularly palisade mesophyll size – were associated with acquisitive strategies. In addition, mesophyll organization, including the ratio of palisade to spongy mesophyll and cell shape, influenced resource acquisition and photosynthetic rate.

**Conclusions:**

Our findings show that conservative strategies predominated in these upper Andean forests; however, a clear acquisitive–conservative gradient persisted and was strongly associated with leaf anatomical traits. These findings indicate that anatomical variation represents a key functional dimension of the LES and provides new insights into its structural basis in tropical montane ecosystems.

## INTRODUCTION

The leaf economics spectrum (LES) provides a conceptual framework for understanding relationships among leaf traits and their role in characterizing plant ecological strategies worldwide (Wright *et al.*, 2004). In general, the traits encompassed within the LES are closely associated with resource acquisition, processing and conservation (Reich, 2014). This spectrum explains ∼75 % of interspecific variation in traits related to carbon gain and nutrient concentration (Wright *et al.*, 2004; Osnas *et al.*, 2013; Díaz *et al.*, 2016) and delineates two fundamental strategies. At one end of the spectrum, species exhibit an acquisitive strategy, characterized by high leaf nutrient content, high capacity for carbon fixation, high respiration rates and shorter leaf lifespan. This is reflected in low leaf mass per area (LMA), high maximum photosynthetic capacity (*A*_max_) and high dark respiration rate (*R*_d_), along with elevated leaf nitrogen content (LNC) and low leaf longevity (Wright *et al.*, 2004; Garnier *et al.*, 2016; Chen *et al.*, 2020). At the opposite end, species follow a conservative strategy, featuring longer leaf lifespans and higher LMA, leaf carbon content (LCC) and carbon to nitrogen ratio (C:N ratio), but lower LNC, *A*_max_ and *R*_d_ (Wright *et al.*, 2004; Garnier *et al.*, 2016; Pan *et al.*, 2020).

The modulation of the LES typically results from internal changes in leaf anatomy (Harrison *et al.*, 2021), including the size of both abaxial and adaxial cuticles and epidermal tissues, hypodermis, palisade and spongy mesophyll, and vascular bundles (Bolhàr-Nordenkampf and Draxler, 1993). These anatomical regions contribute to distinct physiological functions; for instance, the cuticle, hypodermis and epidermis offer protection against biotic and abiotic factors such as insects, microorganisms, pathogens, wind, dehydration and UV-B radiation, while also providing mechanical support and influencing leaf longevity (Bilger *et al.*, 1997; Onoda *et al.*, 2012; Villar *et al.*, 2013; Nakamura *et al.*, 2015; Li *et al.*, 2025). These structures also contribute to overall leaf thickness (LT) (Castillo-Figueroa and Posada, 2025*a*), increasing heat capacity and buffering rapid temperature fluctuations (Vogel, 2009; González *et al.*, 2025). The palisade mesophyll, located beneath the upper epidermis, consists of elongated, tightly packed cells rich in chloroplasts (Terashima and Hikosaka, 1995; Terashima *et al.*, 2011). This structure optimizes light absorption, as it serves as the primary site for converting light energy into chemical energy during photosynthesis (Oguchi *et al.*, 2018). However, thicker palisade cell walls are associated with greater mesophyll resistance to gas diffusion, and consequently lower photosynthetic rates (Onoda *et al.*, 2017). Below the palisade mesophyll, the spongy mesophyll comprises irregularly shaped cells with large air spaces (Terashima *et al.*, 2011). These spaces facilitate the diffusion of gases, such as CO_2_ for photosynthesis and O_2_ as a by-product of carbon fixation and the final acceptor of electrons in respiration (Borsuk *et al.*, 2022). Moreover, the spongy mesophyll enhances light reflection and refraction, increasing the optical path length within the leaf and thereby promoting greater light absorption, an effect known as the ‘detour’ effect (Brodersen and Vogelmann, 2010). Vascular bundles play a key role in the import of water and nutrients to leaves and in the export of carbohydrates (Lucas *et al.*, 2013). Larger vascular bundles are also associated with the ability to withstand environmental stress (Endo *et al.*, 2008) and are important for providing mechanical and structural strength to the leaf (Lucas *et al.*, 2013).

Delving deeper into the anatomical mechanisms underlying resource acquisition in the context of the LES could offer a structural and functional perspective on the generalized covariances driven by selective pressures (Olson, 2019; Harrison *et al.*, 2021). In this regard, previous investigations have suggested that the LES is shaped by fundamental trade-offs between allocation to structural tissues and liquid-phase processes (Shipley *et al.*, 2006; Onoda *et al.*, 2017). This interplay is, to some extent, reflected in the associations among cuticles, epidermis and mesophyll tissues (Onoda *et al.*, 2012; Somavilla *et al.*, 2014; Harrison *et al.*, 2021), as well as in the allometry of cells within leaves (John *et al.*, 2013; Baird *et al.*, 2024). However, we still have a poor understanding of the relationships between foliar and anatomical functional traits related to the LES.

The Andes is the world’s longest continental mountain range, extending nearly 8000 km from Venezuela to Chile (Armenteras *et al.*, 2011; Myster, 2021). The Tropical Andes cover ∼1.5 million square kilometres between 11°N and 23°S, spanning elevations from sea level to over 6000 m (Rodríguez *et al.*, 2013). This region constitutes the largest biodiversity hotspot on Earth, harbouring more than 45 000 plant species (Myers *et al.*, 2000). Within it, Andean mountain forests rank among the most diverse ecosystems worldwide (Pérez-Escobar *et al.*, 2022), characterized by exceptionally high plant β-diversity (e.g. Hurtado-M *et al.*, 2021) and remarkable levels of endemism (Myers *et al.*, 2000). In addition, these forests are globally recognized as strategic ecosystems, providing essential services for water regulation and carbon cycling (Castillo-Figueroa, 2021; Duque *et al.*, 2021; Myster, 2021). In these tropical montane forests, recent studies have revealed a distinctive functional transition in plant communities, marked by shifts in foliar traits towards comparatively conservative strategies, including lower nutrient concentrations, higher LMA and increased leaf toughness and thickness (Homeier *et al.*, 2021; Martínez-Villa *et al.*, 2024). Such adaptations are largely driven by strong environmental filters such as nutrient-poor soils, harsh winds, low temperatures, periodic drought stress, high UV-B radiation and high precipitation (Myster, 2021). Despite this, research on leaf anatomical traits in the Andean flora remains scarce (Carmona *et al.*, 2018; Castillo-Figueroa, 2025), and no study has systematically examined their associations with multiple foliar functional traits linked to the LES. Addressing this gap could provide valuable insights into the mechanisms underpinning plant ecological strategies in tropical Andean montane forests, a region shaped by particular environmental constraints.

In this study, we aimed to understand the underlying functional basis of the LES in upper Andean tropical forests, providing an anatomical perspective on the trade-offs associated with this spectrum. We examined multiple anatomical traits that may be related to mechanisms shaping LES strategies, offering deeper insight into resource allocation within this framework (Harrison *et al.*, 2021). To do so, we analysed correlations between 9 foliar traits derived from the LES and 41 leaf anatomical traits. Our working hypothesis posited that increased thickness in protective and conductive tissues, such as the cuticle, epidermis and vascular bundles, would be positively correlated with conservative whole-leaf traits, including LCC, LMA and the C:N ratio. Conversely, we expected proportionally higher thickness in photosynthetically active tissues, such as the palisade and spongy mesophyll layers, to be positively associated with acquisitive traits like high LNC and *A*_max_. We further discuss potential mechanisms linking leaf anatomical traits to foliar functional traits within the LES framework in upper Andean tropical forests.

## MATERIALS AND METHODS

### Study area

Our study was conducted in the Eastern Colombian Andes, specifically in the Cundiboyacense high plains, a region within the tropical forests of the Andean mountains. This area is heavily influenced by human activities such as agriculture, cattle ranching, urban development and mining (Armenteras *et al.*, 2011; Etter *et al.*, 2021). The research was based on permanent plots established as part of the *Rastrojos* project, initiated in 2013 to monitor biodiversity, ecosystem resilience and carbon cycling along successional gradients in peri-urban upper Andean forests. The *Rastrojos* project includes a total of 36 plots (20 × 20 m) and eight larger plots (50 × 50 m) located around Bogotá (Hurtado-M *et al.*, 2021; Castillo-Figueroa *et al.*, 2023; Castillo-Figueroa and Posada, 2025*b*). For this study, we sampled 63 species from 14 plots (20 × 20 m) distributed at elevations ranging from 2685 to 3150 m. These sites are forest ecosystems representative of the Andean region, all corresponding to the same habitat type (tropical upper Andean montane forest), and are located within private lands or reserves. Sites were selected to capture the dominant environmental and functional conditions within this forest type, while ensuring accessibility and landowner permission. Additional details about the study sites are provided in Castillo-Figueroa (2024*a*, *b*).

### Sampled species

Within the 14 sampled plots, five plant families – Ericaceae, Melastomataceae, Cunoniaceae, Primulaceae and Asteraceae – represent 56 % of all individuals with a basal diameter exceeding 5 cm. The most common genera include *Miconia*, *Weinmannia*, *Cavendishia*, *Myrsine* and *Myrcianthes*, which collectively account for 51 % of all individuals. A total of 63 species, 50 genera and 35 families of shrubs and trees were recorded in the 14 plots (Supplementary Data Table S1), with dominant species such as *Weinmannia tomentosa*, *Cavendishia bracteata*, *Miconia ligustrina*, *Miconia squamulosa* and *Myrcianthes leucoxyla* (Castillo-Figueroa *et al.*, 2023; Castillo-Figueroa, 2024*a*, *b*). To ensure consistency, plant nomenclature was standardized using the International Plant Names Index (https://www.ipni.org/). All the tree and shrub species were selected to capture ecologically meaningful variation relevant to LES-related processes at the community level (Supplementary Data Tables S2 and S3). For example, the key LES traits LMA, *A*_max_ (mass-based) and LNC (mass-based) varied on average by ∼5-fold (53.74–271.38 g m^−2^), 30-fold (7.35–220.73 nmol g^−1^ s^−1^) and 9-fold (4.86–46.01 mg g^−1^), respectively (Supplementary Data Table S3). Thus, the sampled species spanned a substantial fraction of the global LES trait space reported by Wright *et al.* (2004), covering roughly one-third of the global LMA gradient, about half of the *A*_max_ gradient and approximately two-thirds of the LNC gradient. The observed ranges encompass much of the functional variation typical of forest plant communities, indicating adequate coverage of LES-relevant trait space at a regional scale.

### Foliar and anatomical trait measurements

We measured green leaf traits for all 63 species, with three individuals per species. These leaves were collected within the permanent plots from the sunny canopy using a branch pruner, following standardized protocols (Pérez-Harguindeguy *et al.*, 2013). We used previously available measures of leaf photosynthetic capacity (*A*_max mass_, nmol g^−1^ s^−1^; and A_max_  _area_, μmol m^−2^ s^−1^), which were taken with a LICOR (LI-6400XT, LiCor^®^, Lincoln, NE, USA). One-sided leaf area was obtained by scanning the leaves and analysing the resulting images using ImageJ (Schneider *et al.*, 2012, https://imagej.net/ij/). To determine fresh mass (g), leaves were allowed to rehydrate by placing them in plastic bags filled with moist paper for 24–48 h at a low temperature (4 °C) in the dark, following the complete rehydration method proposed by Garnier *et al.* (2001). Each leaf sample was then dried in the oven at 60 °C until constant weight (∼72 h) to determine its dry mass (g). Additional traits, including LMA (g m^−2^) and leaf dry matter content (LDMC, mg g^−1^), were also measured. For chemical traits, an elemental analyser (FlashSmart™, Thermo Fisher Scientific, USA) was used to determine leaf nitrogen content (LNC_mass_, mg g^−1^; LNC_area_, g m^−2^), leaf carbon content (LCC_mass_, mg g^−1^; LCC_area_, g m^−2^) and their ratio (C:N ratio).

To characterize leaf anatomical traits, we measured the epidermis, palisade mesophyll, spongy mesophyll, vascular bundle and air space of all the studied species (Castillo-Figueroa, 2025; Castillo-Figueroa and Posada, 2025*a*). We collected three leaves for each species, one from each individual in the permanent plots, and subsequently obtained three rectangular sections (measuring 1 cm × 0.5 cm) that included the midrib and a portion of the lamina. These sections were fixed in FAA solution (50 mL 38 % formalin, 50 mL glacial acetic acid and 900 mL 70 % ethanol) (He *et al.*, 2018). These leaf tissues underwent gradual dehydration in an ethanol series (ranging from 50 to 100 %) and were then infiltrated with hot paraffin (He *et al.*, 2018; Harrison *et al.*, 2021). Subsequently, transverse sections of the leaves were obtained using a Leica RM2255 microtome to create a flat surface for the subsequent estimation of tissue fractions. The anatomical sections of the leaves had a thickness of 5–7 µm. Lignified tissues were stained with safranin, while non-lignified tissues were stained with toluidine blue. After staining, the sections were mounted on slides (Bancroft and Cook, 1984; He *et al.*, 2018).

Following this, we took photographs of the leaves using a reflected (episcopic) light illumination microscope (Leica DM750) equipped with a digital camera (Leica MC170 HD) and a 10× objective lens. Calibration was performed using a Nikon micrometer (MBM 11 100 stage micrometer type A) with a total length of 1 mm and graduations of 0.01 mm (i.e. 10 μm). For each individual, we took 10 focused images of different leaf segments, resulting in a total of 30 images per species (Castillo-Figueroa, 2025; Castillo-Figueroa and Posada, 2025*a*).

**Fig. 1.**
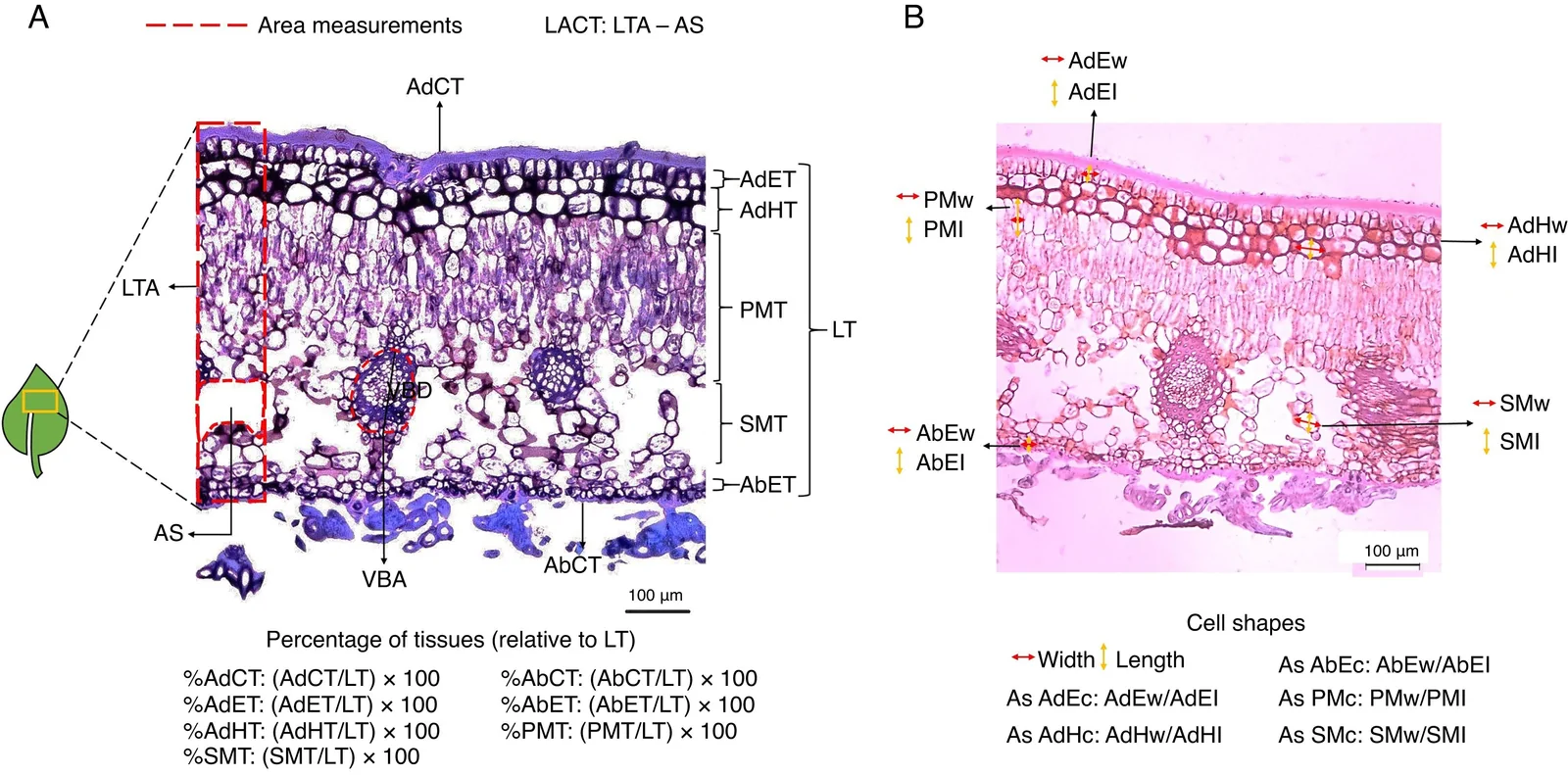
Light microscopy image of leaf transverse profile in *Oreopanax bogotensis* showing the different anatomical traits measured in this study. (A) Measurements on tissue. AdCT, adaxial cuticle thickness (µm); AbCT, abaxial cuticle thickness (µm); AdET, adaxial epidermis thickness (µm); AbET, abaxial epidermis thickness (µm); AdHT, adaxial hypodermis thickness (µm); PMT, palisade mesophyll thickness (µm); SMT, spongy mesophyll thickness (µm); AS, air space (µm^2^); LTA, leaf total area (µm^2^); LACT, leaf area cell tissue (µm^2^) VBA, vascular bundle area (µm^2^); VBD, vascular bundle diameter (µm); LT, leaf thickness (µm). (B) Measurements on cell types. AdEw, adaxial epidermis cell width (µm); AdEl, adaxial epidermis cell length (µm); AdHw, adaxial hypodermis cell width (µm); AdHl, adaxial hypodermis cell length (µm); PMw, palisade mesophyll cell width (µm); PMl, palisade mesophyll cell length (µm); SMw, spongy mesophyll cell width (µm); SMl, spongy mesophyll cell length (µm); AbEw, abaxial epidermis cell width (µm); AbEl, abaxial epidermis cell length (µm). Tissue ratios were derived for adaxial epidermis/palisade mesophyll thickness (AdET/PMT), adaxial epidermis/spongy mesophyll thickness (AdET/SMT), palisade/spongy mesophyll thickness (PMT/SMT), adaxial/abaxial epidermis thickness (AdET/AbET) and adaxial/abaxial cuticle thickness (AdCT/AbCT).

For each anatomical leaf image, we measured the thickness of the cuticle, hypodermis (when present), epidermis, palisade mesophyll, spongy mesophyll and vascular bundles in each cross-section using ImageJ software (Fig. 1). From these measurements, we also calculated the percentage contribution of each anatomical trait relative to the total LT. To estimate the percentage of air spaces, we selected ten rectangles, each measuring 100 µm in width and spanning the total LT, and quantified the area occupied by leaf tissue versus air space. These sections were enhanced for colour and contrast, then processed in the software ImageJ, converting cell tissues to black and air spaces to white (Harrison *et al.*, 2021). Additionally, we derived ratios for different anatomical traits, including adaxial epidermis/palisade mesophyll thickness (AdET/PMT), adaxial epidermis/spongy mesophyll thickness (AdET/SMT), palisade/spongy mesophyll thickness (PMT/SMT), adaxial/abaxial epidermis thickness (AdET/AbET) and adaxial/abaxial cuticle thickness (AdCT/AbCT). For cell morphology, we measured the length and width of ten randomly selected cells from the adaxial and abaxial epidermis, palisade mesophyll and spongy mesophyll, using these dimensions to calculate cell aspect ratios (As: width/length). Higher aspect ratios indicate more circular or squared cells, while lower ratios reflect cylindrical or rectangular shapes. All anatomical trait measurements were averaged at the species level (Castillo-Figueroa, 2025; Castillo-Figueroa and Posada, 2025*a*). Abbreviations of all anatomical traits can be found in Supplementary Data Table S4 and the legend of Fig. 1.

### Statistical analysis

All the statistical analyses were performed in PAST 4.0 and JASP 0.18.1.0. We examined Spearman correlations between the functional traits measured given that the Mardia test for multivariate normality indicated a significant deviation from normality (*P* < 0.0001). These correlations were presented in four separate plots: absolute measurements, tissue percentages, ratios and cell shapes.

To assess how leaf anatomical traits related to the ‘traditional’ whole-leaf traits, we conducted a principal component analysis (PCA) using anatomical and foliar traits as variables, with species as data points (*n* = 63). Prior to the ordination analysis, all trait variables were standardized using *Z*-scores (i.e. each value was centred by subtracting the mean and scaled by dividing by the standard deviation) to ensure comparability across scales and units. This standardization was necessary to avoid the dominance of variables with larger numerical ranges in the PCA (Jolliffe and Cadima, 2016). Data points were scaled by 1/√*d_k_* and the biplot eigenvectors by √*d_k_*, where *d_k_* is the number of dimensions retained in the ordination, according to the correlation biplot of Legendre and Legendre (1998).

In the PCA we used tissue proportions and mesophyll ratios rather than absolute tissue thickness, because relative values account for differences in overall leaf size and better represent functional allocation among leaf tissues. The only absolute traits retained were LT and vascular bundle diameter, as they also provide meaningful insight into ecological strategies. For the PCA, we included only the percentage of adaxial epidermis and cuticle thickness, due to their strong correlation with their abaxial counterparts (*P* < 0.0001; ρ ≥ 0.65). The percentage of hypodermis thickness was excluded because it was absent in more than half of the species (35 species, 55.5 %; Supplementary Data Table S2).

Finally, relationships between LMA and a subset of key foliar and anatomical traits were assessed using reduced major axis (RMA) regressions to synthesize the main associations across different functional dimensions. The selected traits were chosen because they capture central aspects of the LES (Harrison *et al.*, 2021; Ni *et al.*, 2022), including resource-use strategy (i.e. C:N ratio and *A*_max_  _mass_) and underlying anatomical structure (i.e. vascular bundle diameter and palisade mesophyll thickness), and show consistently strong associations with LMA observed in the correlation analyses (Supplementary Data Tables S5–S8), ensuring representation of the main acquisitive–conservative gradient. The use of RMA regression was appropriate because both variables are subject to measurement error and the relationships were interpreted as functional associations rather than strictly predictive models. Coefficients of determination (*R*^2^) and associated *P*-values were obtained for each regression.

## RESULTS

### Foliar and anatomical traits

We found a substantial variation in all the foliar and anatomical traits measured (Supplementary Data Tables S2 and S3). Foliar traits showed wide ranges in their structural and functional attributes, including LMA, LDMC, carbon and nitrogen contents and photosynthetic capacity (Supplementary Data Table S3). Anatomical traits also exhibited high variability, particularly in tissue thickness (e.g. palisade and spongy mesophyll), vascular dimensions and air space (Supplementary Data Table S2).

### Correlations between anatomical traits and foliar traits

**Fig. 2.**
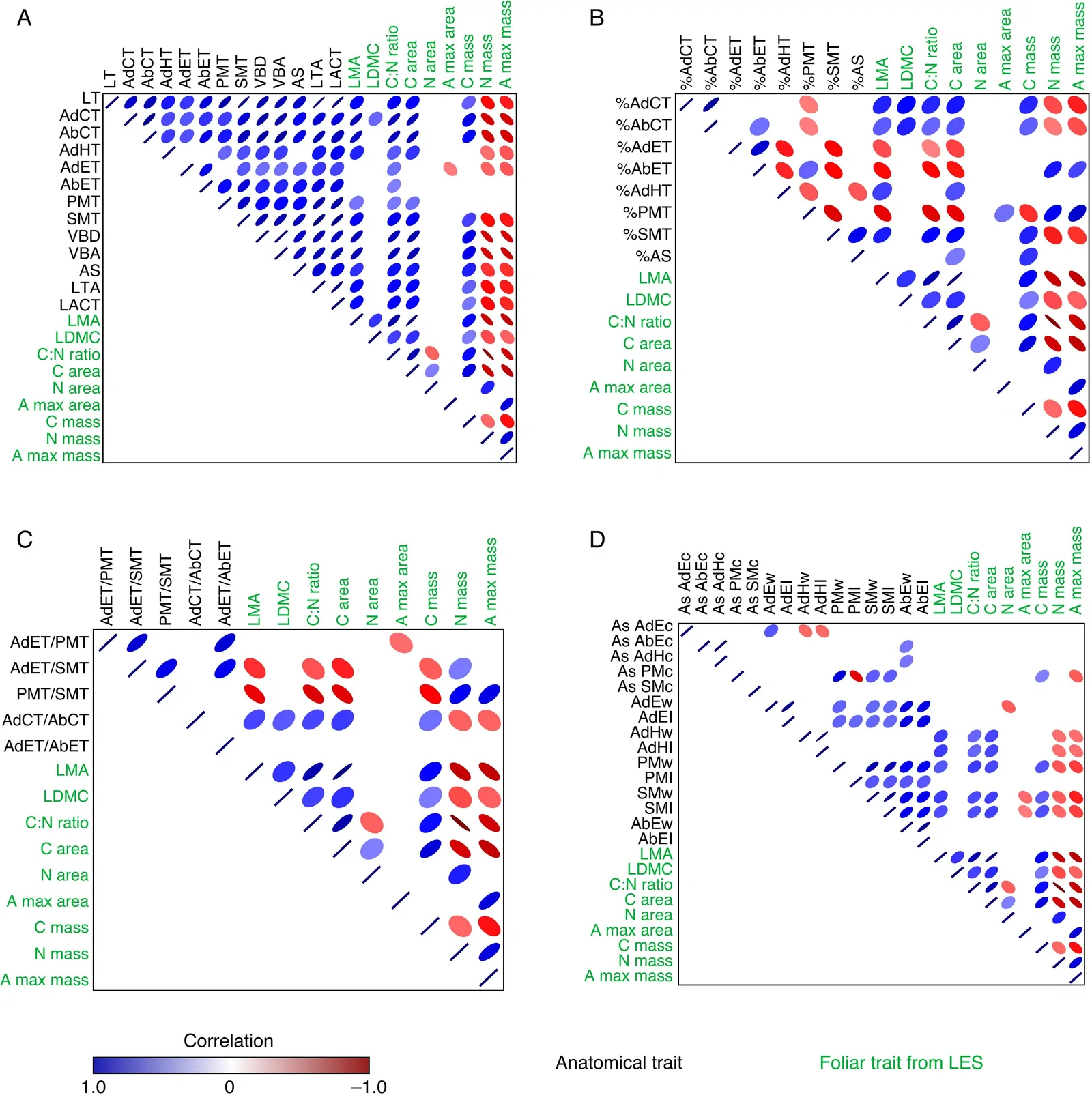
Spearman correlations between anatomical traits (black) and foliar traits (green) considering (A) absolute measurements, (B) percentage of the tissues, (C) ratios between tissues and (D) cell length, width and aspect ratio for each tissue. Key colours show the correlation coefficient and its corresponding colour gradient from −1 (negative correlation, red colours) to 1 (positive correlation, blue colours). The colour intensity and size of the ellipses are proportional to the correlation coefficients, where more diffuse and larger ellipses signify weaker correlation strengths. When the correlation is non-significant (*P* > 0.05) it is represented with a blank. Abbreviations of all anatomical traits can be found in the legend of Fig. 1 and Supplementary Data Table S4.

Relationships among foliar traits were consistent with the LES. Traits associated with resource conservation (e.g. high LMA and C:N ratio) were negatively related to nitrogen content and photosynthetic capacity. Leaf thickness showed positive relationships with the thickness of all anatomical tissues. Thicker leaves were linked to whole-leaf traits indicative of conservative strategies, including higher LMA, C:N ratio and carbon content (LCC_area_ and LCC_mass_) and lower nitrogen content (LNC_mass_) and photosynthetic capacity (*A*_max mass_). No significant relationships were found between LT and LDMC or area-based traits such as LNC_area_ and *A*_max area_ (Fig. 2).

Photosynthetic capacity per unit mass (*A*_max mass_) showed a consistent pattern of association with both foliar and anatomical traits. It was positively related to nitrogen content (LNC_mass_) and area-based photosynthesis (*A*_max area_), but negatively associated with whole-leaf traits linked to structural investment and carbon accumulation, such as LMA, C:N ratio, LCC_mass_ and LCC_area_. At the anatomical level, *A*_max mass_ decreased with increasing thickness and size of multiple leaf tissues, including cuticles, epidermis, hypodermis, spongy mesophyll layers and vascular structures (e.g. AdCT, AdET, SMT, vascular bundle diameter (VBD)), as well as with greater air space and larger cell dimensions (e.g. air space (AS), adaxial hypodermis cell width (AdHw), adaxial hypodermis cell length (AdHl)). In contrast, it increased with traits reflecting a greater relative investment in palisade mesophyll (e.g. percentage PMT (%PMT) and PMT/SMT) (Fig. 2).

Overall, these patterns indicate a clear trade-off between structural investment and photosynthetic performance, with thicker and more structurally complex leaves exhibiting lower mass-based photosynthetic capacity, whereas greater relative investment in palisade mesophyll is associated with higher photosynthetic rates. Area-based traits exhibited weaker and less consistent relationships with anatomical variables than mass-based traits. Notably, LDMC showed only a few significant associations with anatomical traits, with most relationships being non-significant (*P* > 0.05) (Fig. 2). Full correlation coefficients and associated *P*-values for absolute measurements (Supplementary Data Table S5), tissue percentages (Supplementary Data Table S6), ratios (Supplementary Data Table S7) and cell shape traits (Supplementary Data Table S8) are provided in the Supplementary Data.

### Functional variation based on leaf anatomical and foliar traits

**Fig. 3.**
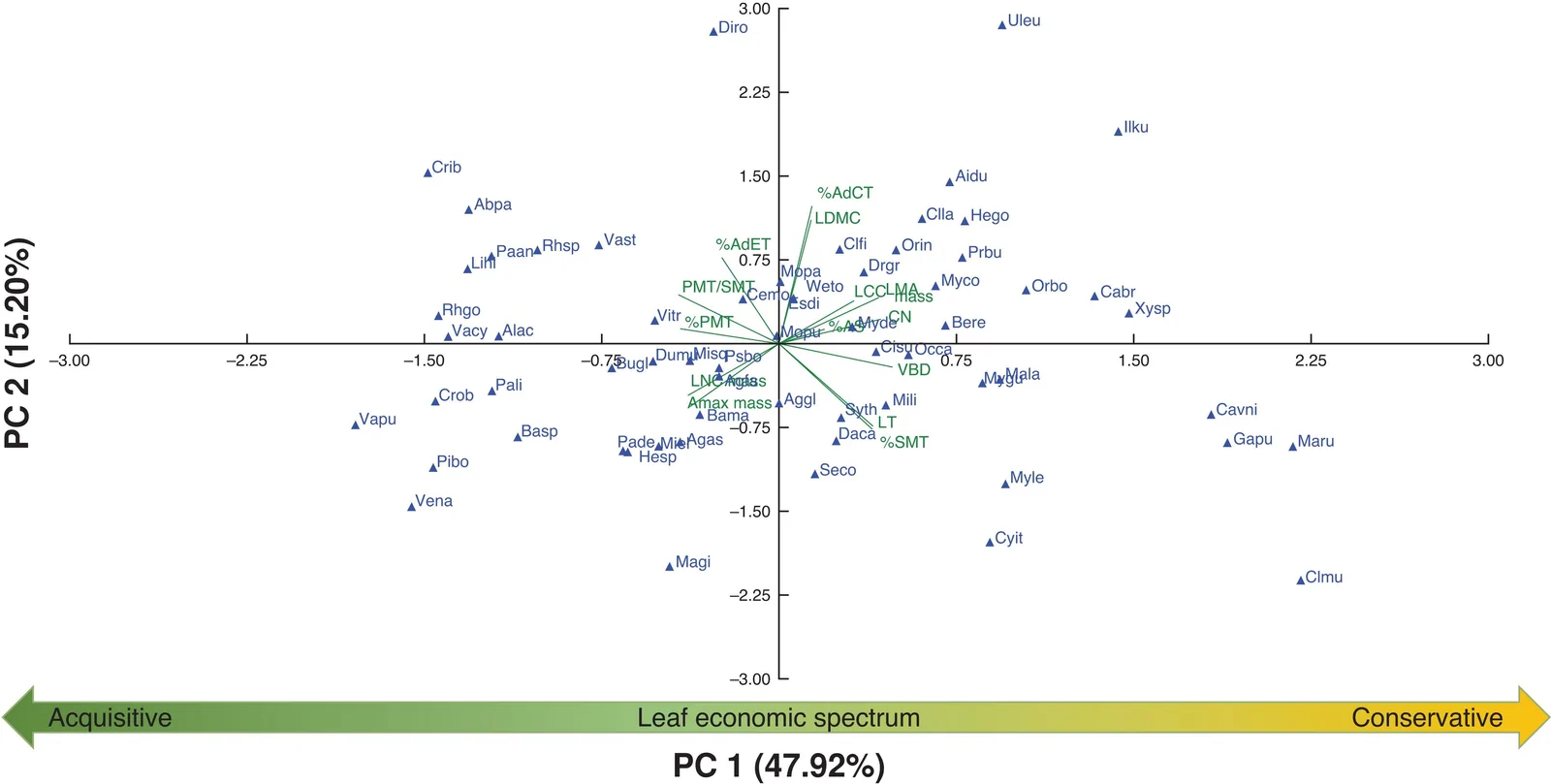
Principal component analysis between foliar and anatomical traits. Green lines indicate the traits: percentage adaxial cuticle thickness (%AdCT), percentage adaxial epidermis thickness (%AdET), percentage palisade mesophyll thickness (%PMT), percentage spongy mesophyll thickness (%SMT), air space (AS, µm^2^), vascular bundle diameter (VBD, µm), leaf thickness (LT, µm), palisade/spongy thickness ratio (PMT/SMT), *A*_max mass_ (*A*_max_, nmol g^−1^ s^−1^), leaf mass per area (LMA, g m^−2^), leaf nitrogen content (LNC_mass_, mg g^−1^), leaf carbon content (LCC_mass_, mg g^−1^), carbon/nitrogen ratio (CN) and leaf dry matter content (LDMC, mg g^−1^). Blue triangles represent the 63 Andean species, and the letters correspond to species acronyms listed in Supplementary Data Table S2.

The PCA revealed a clear functional gradient consistent with the LES. The first axis (47.92 % of variance) separated species with high structural and carbon investment (high LMA, LT, VBD, LCC_mass_, C:N ratio) from those with higher nitrogen content and photosynthetic capacity (high LNC_mass_ and *A*_max mass_). Anatomical traits such as mesophyll thickness and vascular dimensions contributed strongly to this gradient, linking leaf structure with functional strategies. The second axis (15.20 %) captured variation in tissue allocation, particularly the relative investment in palisade versus spongy mesophyll (e.g. %PMT, percentage SMT (%SMT), PMT/SMT), indicating additional axes of anatomical differentiation among species (Fig. 3, Supplementary Data Fig. S1).

**Fig. 4.**
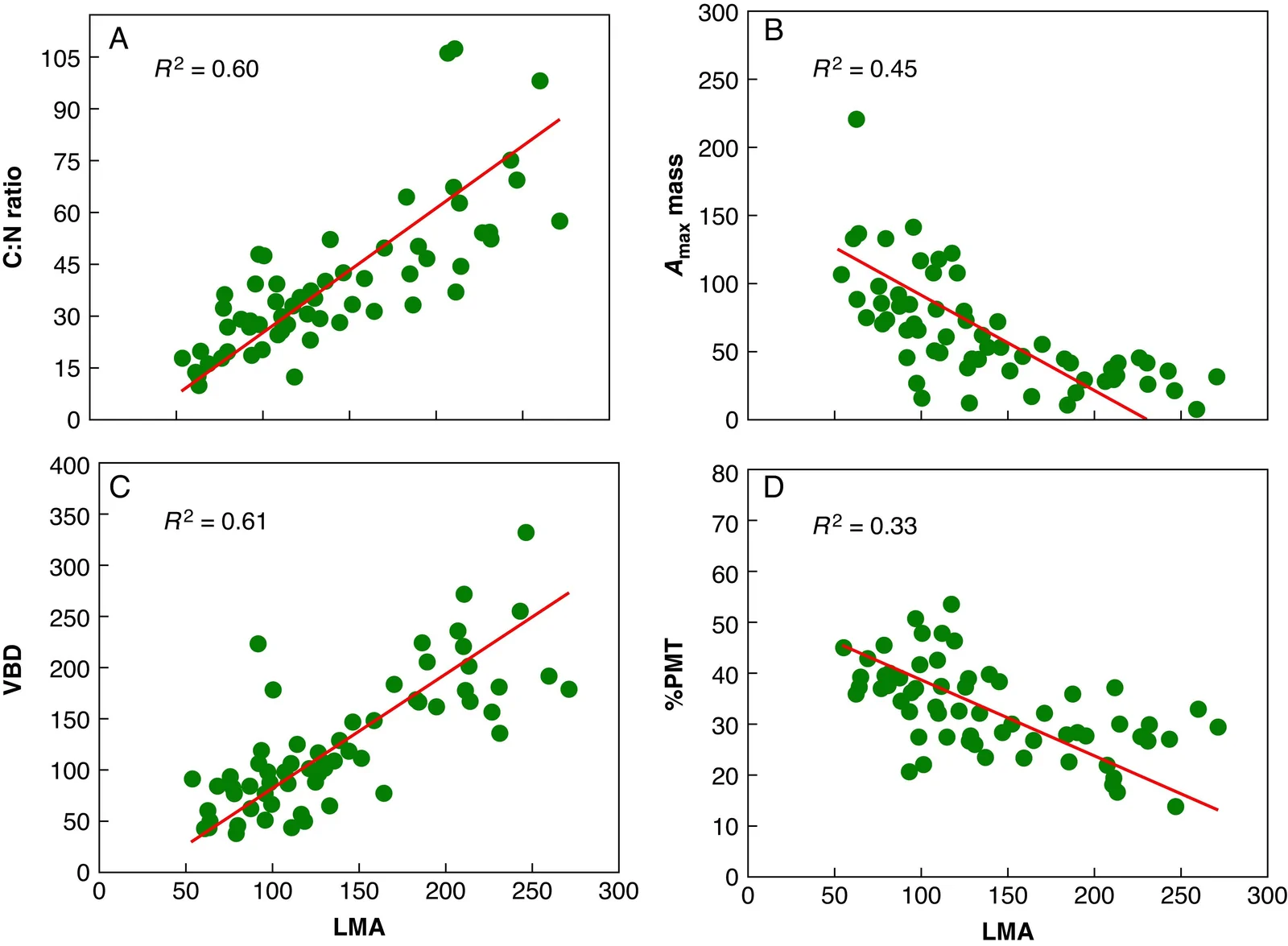
Relationships between LMA and key foliar and anatomical traits. Foliar traits exhibit expected patterns of the LES, while anatomical traits reveal the structural basis underpinning these relationships. RMA regressions show the relationships between LMA and (A) C:N ratio, (B) *A*_max mass_, (C) vascular bundle diameter and (D) percentage palisade mesophyll thickness. Each green point represents a species. Red lines indicate statistically significant relationships (*P* < 0.05).

The patterns revealed by the PCA were further supported by RMA regressions, which showed strong relationships between key traits of the LES and representative foliar and anatomical traits. Specifically, LMA was positively related to the C:N ratio (*P* < 0.0001; *R*^2^ = 0.60; Fig. 4A) and negatively related to *A*_max mass_ (*P* < 0.0001; *R*^2^ = 0.45; Fig. 4B). Among anatomical traits, LMA was positively associated with vascular bundle diameter (*P* < 0.0001, *R*^2^ = 0.61; Fig. 4C) and negatively associated with %PMT (*P* < 0.0001, *R*^2^ = 0.33; Fig. 4D). Together, these results indicate that the structural organization of leaf tissues underpins the observed LES relationships, linking anatomical investment with variation in foliar function across species.

## DISCUSSION

This study reveals the relationships between leaf anatomy and distinct whole-leaf morpho-physiological traits associated with the LES in upper Andean forest species. We found that larger protective and conductive tissues are linked to conservative strategies, while the relative size of the palisade mesophyll is associated with acquisitive strategies. Additionally, the palisade-to-spongy mesophyll ratio and cell shape within these tissues are closely related to traits that underpin acquisition–conservation strategies in plants. Our results also indicate that even under strong environmental filtering typical of upper Andean tropical forests, leaf anatomical variation sustains a functional gradient consistent with the LES, rather than concentrating into a single conservative syndrome. Overall, our study highlights the mechanistic role of leaf anatomical traits in shaping plant functional strategies, providing new insights into how Andean species balance resource acquisition and conservation.

### Leaf anatomy and the LES

Our research reveals that leaf anatomy provides a mechanistic understanding of the underlying basis of the LES given that different tissues are related to resource acquisition and conservation strategies as predicted in the fast–slow continuum (Reich, 2014). In this sense, we support our hypothesis that larger protective tissues (i.e. cuticles and epidermis) and conductive tissues (i.e. diameter/area of the vascular bundles) are more closely associated with conservative whole-leaf traits, characterized by high structural investment, as reflected in higher LMA and LCC. In contrast, the relative size of the palisade mesophyll and the palisade/spongy ratio are strongly linked to acquisitive leaf traits distinguished by greater nitrogen content and higher leaf photosynthetic rates (Figs 2 and 3), which is consistent with the fact that the palisade mesophyll does most of the carbon fixation in sun leaves of woody species.

In terms of protective tissues, cuticle has been suggested as a key structure in the carbon economy and as giving added mechanical strength (Onoda *et al.*, 2012; Li *et al.*, 2025). Besides providing protection against pathogens, thicker cuticles and epidermis may also help filter harmful UV-B radiation, regulate CO_2_ diffusion through the stomata and reduce water loss, which are features typically associated with conservative strategies exhibiting low *A*_max mass_ (Verboven *et al.*, 2015; He *et al.*, 2018). Moreover, larger vascular bundles can help maintain the structure of leaf tissues undergoing high water loss (Leroux, 2012; Galviz and Valerio, 2021), and increase the strength of leaves to prevent mechanical damage from biotic (e.g. herbivores) and abiotic factors (e.g. hail). Larger vascular bundles can also be related to better water transport, which may help in sustaining transpiration for longer periods of time, yet this depends on the diameter of the vessels, which largely determines hydraulic conductivity (Sack and Holbrook, 2006).

Conversely, in the other extreme of the spectrum, we found that proportionally thicker palisade mesophyll, as well as a high proportion of this tissue in relation to spongy tissue, was positively related to *A*_max mass_ and LNC_mass_ (Figs 2 and 3). This could be explained by the fact that thicker palisade tissues contain the most chloroplasts, proteins and chlorophyll in the leaf (Terashima *et al.*, 2011; He *et al.*, 2018; Oguchi *et al.*, 2018; Borsuk and Brodersen, 2019; Harrison *et al.*, 2021). Overall, a large fraction (70–80 %) of leaf nitrogen is allocated to chloroplasts, and particularly to Rubisco, which can contribute ∼40 % of chloroplast nitrogen (Evans and Terashima, 1987; Terashima and Hikosaka, 1995; Onoda *et al.*, 2017).

The potential mechanisms related to anatomical trait relationships and trade-offs that underpin the LES are still under debate. In this regard, Shipley *et al.* (2006) suggested that the LES is governed by fundamental trade-offs between allocation to structural tissues and to liquid-phase (i.e. metabolic) processes, which are reflected in the ratio between cell volume and cell wall volume. In addition, Onoda *et al.* (2017) established two non-mutually exclusive hypotheses related to the relationships between *A*_max mass_ and LNC. The first hypothesis, named ‘nitrogen allocation trade-off’, states that a higher investment of nitrogen in cell walls rather than photosynthetic components of the mesophylls is associated with higher LMA, greater physical strength and longer leaf lifespan, at the cost of lower *A*_max mass_ (Onoda *et al.*, 2017). The second hypothesis, known as the ‘CO_2_ diffusion limitation’ hypothesis, proposes that increased investment in mesophyll tissues may enhance photosynthetic capacity by increasing chloroplast number and internal surface area available for CO_2_ diffusion, while simultaneously imposing diffusional constraints by reducing mesophyll conductance and CO_2_ availability at carboxylation sites due to increased mesophyll cell wall thickness, which increases resistance to CO_2_ diffusion (Onoda *et al.*, 2017). The net effect on photosynthesis therefore reflects a balance between enhanced biochemical capacity and increased diffusion resistance. Although our study did not explore cell structures as such, we found that high investments in protective tissues support a nitrogen allocation trade-off. However, we do not discard the CO_2_ diffusion limitation hypothesis, as internal CO_2_ diffusion within the leaf is strongly constrained by mesophyll cell wall thickness, which represents one of the main structural resistances to CO_2_ diffusion, in addition to stomatal regulation of gas exchange, although these anatomical traits were not measured in our study.

Even though spongy mesophyll enhances carbon assimilation by improving gas transportation and photosynthesis (Terashima *et al.*, 2011; Borsuk *et al.*, 2022), in our study *A*_max mass_ decreased with increasing spongy tissue thickness, contrary to what was expected. This suggests that species with larger spongy tissue also exhibit greater overall leaf thickness and structural investment, leading to lower mass-based photosynthetic capacity. This pattern likely reflects a functional trade-off in which greater investment in spongy mesophyll may support a more efficient and stable carbon gain rather than increasing *A*_max mass_. Specifically, a thicker spongy mesophyll may (1) improve light capture efficiency through greater diffraction in more conservative leaves, compensating for their lower *A*_max mass_; (2) increase the internal gas volume, creating a buffer against rapid fluctuations in light intensity and thereby helping to maintain photosynthesis during sudden increases in radiation; and (3) expand the internal CO_2_ exchange surface, compensating for the greater diffusion resistance imposed by the thicker palisade mesophyll cell walls.

### Anatomical traits and photosynthesis

Surprisingly, we found that the shape of cells was also related to *A*_max mass_ (Fig. 2). In particular, we found that species with longer and thinner palisade cells had higher *A*_max mass_ than cells with more rounded shape. It is likely that this cell type increases the cell surface, enabling a larger area to come in contact with light, thus optimizing photosynthesis. Building on previous work showing links between palisade cell geometry, light directional quality and photosynthetic performance (Borsuk *et al.*, 2025), this study further examines the relationship between palisade cell aspect ratio and photosynthetic capacity in a different ecological context. Furthermore, both longer and wider spongy cells were negatively related to *A*_max mass_, suggesting a reduction in the total surface area exposed to intercellular air spaces, thereby limiting CO_2_ diffusion efficiency and ultimately constraining photosynthesis (Evans *et al.*, 2009; Terashima *et al.*, 2011); yet thicker spongy mesophylls may partially compensate for this effect at the tissue level by increasing the overall surface area available for gas exchange.

Importantly, we observed a negative relation between *A*_max mass_ and hypodermis thickness, as well as the length and width of its cells. The hypodermis, situated immediately below the epidermis and above the palisade layer, is characterized by hyaline contents and thin walls (Nakamura *et al.*, 2015). This tissue plays a crucial role in providing structural support (Lee, 1953), water storage (Reginato *et al.*, 2009), thermal tolerance (González *et al.*, 2025) and the regulation of photosynthetic efficiency (Horner, 2012). Notably, the presence of a well-developed hypodermis is a recurrent anatomical feature in many woody species from tropical Andean montane forests (44.5 % of the 63 species in this study), yet it is relatively uncommon in lowland mesophytic tropical forests (Turner *et al.*, 1995; Boeger *et al.*, 2004). This anatomical differentiation likely reflects adaptation to the distinctive environmental conditions of montane systems, including high solar radiation, large diurnal temperature fluctuations and persistent cloud cover (Ruiz *et al.*, 2012; Castillo-Figueroa, 2025; González *et al.*, 2025). Under these conditions, the hypodermis may function primarily as a protective and buffering tissue rather than as a structure optimizing carbon gain. Consistent with this interpretation, some authors have also suggested that the hypodermis could reduce light penetration within the leaf to some degree, which could help explain why leaves with thicker hypodermis have lower photosynthetic capacity (Nakamura *et al.*, 2015). Moreover, the hypodermis may function as a protective tissue against excessive solar UV-B radiation (Horner, 2012; Nakamura *et al.*, 2015; González *et al.*, 2025), which is likely a key environmental filter in upper Andean tropical mountains. Although this hypothesis has remained largely untested from a physiological perspective, our results show that a thicker hypodermis layer is related to conservative leaves of low photosynthetic capacity (Fig. 2), which are well protected, as reflected by multiple layers of longer and wider cells.

Finally, we found a positive relation between the proportion of abaxial epidermis and *A*_max_  _mass_ (Fig. 2). In woody species, the abaxial epidermis usually concentrates a higher density of stomata where gas exchange occurs between the leaf and the atmosphere and operates independently of the adaxial surface (Driscoll *et al.*, 2006; Wall *et al.*, 2022). Furthermore, the abaxial epidermis acts as a key interface for gas exchange, facilitating both CO_2_ diffusion and transpiration (Wall *et al.*, 2022). This functional role becomes particularly relevant at higher elevations, where the partial pressure of CO_2_ decreases (Körner, 2007). Under these conditions, greater investment in the abaxial epidermal compartment may contribute to maintaining CO_2_ diffusion into internal leaf spaces through stomatal regulation (Körner and Diemer, 1987; Liu *et al.*, 2020), partially offsetting atmospheric diffusion limitations.

### Trait decoupling and differences between mass- and area-based relationships

Not all relationships between anatomical and leaf functional traits were significant (Fig. 2, Supplementary Data Tables S5–S8). While multiple patterns aligned with expectations based on the LES, others were weak or absent. This heterogeneity suggests that trait coordination is not uniform and that different dimensions of leaf structure and function may operate somewhat independently. In this context, non-significant relationships are informative, as they reveal limits to the coupling between anatomical traits and functional strategies, highlighting the multidimensional nature of the LES.

Consistent with this pattern, we found that LT showed strong and coordinated relationships with multiple structural and functional traits. Specifically, LT was positively related to the thickness of all tissue layers, as well as to LCC, LMA and the C:N ratio, while it was negatively related to *A*_max mass_ and LNC_mass_. However, despite this clear coordination, LT and most anatomical traits, except for cuticles, were unrelated to LDMC. This suggests that LDMC may not be influenced by variations in tissue thickness. Although LDMC is a key trait associated with plant ecological strategies, often reflecting a trade-off between rapid growth and resource acquisition on one end of the LES, and efficient resource conservation in well-protected tissues on the other (Wilson *et al.*, 1999; Garnier *et al.*, 2001, 2016), it appeared orthogonal to the resource acquisition axis (Fig. 3). Additionally, recent studies in Andean tropical montane forests have found that LDMC is unrelated to litter decomposability and is also orthogonal to this axis (Castillo-Figueroa *et al.*, 2025; Castillo-Figueroa and Posada, 2025*a*), despite previous evidence highlighting its importance in determining litter quality and decomposition (de la Riva *et al.*, 2019; Wang *et al.*, 2025). One might expect that the presence of a hypodermis may simultaneously increase leaf water-holding capacity and promote longer leaf longevity; these contrasting effects could offset each other in their influence on LDMC, as longer leaf lifespan tends to increase LDMC whereas higher water content tends to decrease it. However, our results suggest that the hypodermis is more strongly associated with leaf longevity and structural investment than with water-holding capacity. Specifically, the hypodermis was positively related to LMA, C:N ratio and LCC_area_ (Fig. 2), all of which are traits commonly linked to structural reinforcement and longer leaf lifespan (Wright *et al.*, 2004). In contrast, the hypodermis thickness showed no relationship with LDMC (Fig. 2) or leaf water content (*P* = 0.34; ρ = −0.12; data not shown). Together, these patterns indicate that, in our study system, the hypodermis likely contributes more to leaf longevity than to water storage, which is reasonable given the consistently high moisture conditions of Andean forests. Consequently, the lack of correlation between LDMC and anatomical traits is likely driven by other mechanisms.

Our results also showed that high LMA correlated negatively with LNC_mass_, consistent with several studies conducted worldwide (Wyka *et al.*, 2013; Díaz *et al.*, 2016; Pan *et al.*, 2020; Ji *et al.*, 2021). Moreover, LMA was primarily driven by larger cell and tissue sizes (Fig. 2), as suggested by the Exhaustive Anatomy and Composition of Tissues (EXACT) approach (John *et al.*, 2017). Determining the anatomical basis of LMA is important, as this trait is part of a broader suite of interconnected functional traits that collectively shape plant ecological performance (Poorter *et al.*, 2009) through both metabolic and structural leaf functions (Osnas *et al.*, 2013, 2018; Katabuchi *et al.*, 2025). LMA also plays a central role in mechanical resistance (Onoda *et al.*, 2012) and is crucial for defining plant ecological strategies (Reich, 2014; Garnier *et al.*, 2016). Even though anatomical traits were strongly related to LMA (Fig. 4), this trait may also be influenced by the chemical composition of leaves, including lignin, structural and non-structural carbohydrate and soluble phenolic and protein concentrations, as other studies suggest (Poorter *et al.*, 2009; Wyka *et al.*, 2013; Wang *et al.*, 2021, 2025).

Strikingly, we found that most of the relationships between foliar traits and leaf anatomy were based on mass units rather than area units (Fig. 2). Mass-based traits inherently integrate internal tissue density and thickness, whereas area-based traits are more strongly influenced by leaf surface expansion. This suggests that structural and biochemical investments are more directly linked to anatomical traits associated with mass and internal resource storage, such as tissue thickness (Niinemets, 1999; Wright *et al.*, 2004). In contrast, area-based traits integrate multiple dimensions of leaf function related to external factors, which may weaken the direct influence of specific anatomical traits (Poorter *et al.*, 2009; Onoda *et al.*, 2011). Previous work has shown that differences in scaling between mass- and area-based measurements can obscure relationships for area traits (Poorter *et al.*, 2009; Westoby *et al.*, 2002; Osnas *et al.*, 2013) and that these relationships may shift across and within species (Katabuchi *et al.*, 2025). Indeed, while mass-based traits are often more informative across species (Wright *et al.*, 2004), area-based traits tend to better capture variation within plants (Anderegg *et al.*, 2018; Osnas *et al.*, 2018). While our analyses focused on interspecific patterns, they consistently highlighted the stronger role of mass-based traits, contrasting with some previous findings (Osnas *et al.*, 2018; Katabuchi *et al.*, 2025). Still, our study does not address intraspecific variation, which is key to testing the consistency of these associations. Future work should examine how anatomical traits expressed in area or leaf density units interact with environmental conditions and whether their links to foliar traits vary across organizational scales, both among and within species, to clarify why area-based associations appear weaker in Andean mountain forests.

### Conclusions

Leaf anatomy serves as one of the mechanistic foundations for the LES. In this study, we identified correlations between anatomical structures and diverse foliar traits that are aligned with both acquisitive and conservative plant ecological strategies. Specifically, thicker tissues, encompassing protective, mechanical and conductive functions like cuticles, epidermis, hypodermis and vascular bundles, are correlated with conservative strategies. Conversely, thicker palisade mesophylls are linked to acquisitive strategies. Although these patterns broadly align with general LES theory, our results demonstrate that even in plant communities strongly shaped by environmental filtering and dominated by conservative strategies, such as the upper Andean tropical forests, a clear functional gradient between acquisitive and conservative strategies persists. This indicates that, rather than collapsing into a single conservative syndrome, leaf anatomical variation reflects a constrained yet ecologically meaningful expression of the LES, supporting the coexistence of multiple functional strategies under harsh montane conditions. We propose that this persistence reflects the coexistence of multiple viable anatomical configurations that balance carbon acquisition, tissue protection and leaf longevity under different montane constraints, including reduced atmospheric CO_2_ partial pressure, high radiation and pronounced thermal variability. Under such conditions, selection may limit the range of trait values but still maintain functional differentiation among species, allowing acquisitive and conservative strategies to coexist within a narrowed LES space. Overall, this study contributes valuable insights into the anatomical basis underlying the LES in upper Andean tropical forests.

## Supplementary Material

mcag131_Supplementary_Data

## SUPPLEMENTARY DATA

Supplementary data are available at are openly available in the Open Science Framework repository at https://osf.io/wjr5t/overview. Table S1: list of the 63 plant species sampled in this study, with their respective taxonomic families and life form. Table S2: descriptive statistics from the leaf anatomical traits of the 63 plant species studied. Table S3: descriptive statistics for the foliar traits from the LES of the 63 plant species studied. Table S4: abbreviations and units of the leaf functional traits analysed. Table S5: correlation matrix between absolute anatomical measurements and foliar traits within the LES, showing Spearman’s ρ coefficient. Table S6: correlation matrix between percentages for anatomical measurements and foliar traits within the LES, showing Spearman’s ρ coefficient. Table S7: correlation matrix between anatomical ratios and foliar traits within the LES, showing Spearman’s ρ coefficient. Table S8: correlation matrix between cell shape traits and foliar traits within the LES, showing Spearman’s ρ coefficients. Figure S1: PCA including foliar and anatomical traits showing correlations between each variable and the scores of the first and second axes of the PCA.
