# Supplementary material for: Anatomical and physiological trade-offs underlying the leaf economics spectrum in tropical Andean montane forests
Source: Ann Bot. 2026 May 14;138(4):1118–29. doi: 10.1093/aob/mcag131 (PMC13595238; doi:10.1093/aob/mcag131)
Supplement: Supplementary file 1 [file mcag131_supplementary_data.docx]

Supporting information

**Anatomical and physiological trade-offs underlying the leaf economics spectrum in tropical Andean montane forests**

Dennis Castillo-Figueroa^1*^, Juan M. Posada^1^

1. School of Sciences and Engineering, Universidad del Rosario, Kr 26 # 63B-48, Bogotá, Colombia.

Corresponding author: [dennis.castillof@gmail.com](mailto:dennis.castillof@gmail.com)

**Table S1.** List of the 63 plant species sampled in this study, with their respective taxonomic families and life form.

| **FAMILY/**Species | **Life form** |
| --- | --- |
| **AQUIFOLIACEAE Bercht. & J.Presl** |  |
| *Ilex*  *kunthiana* Triana | Tree |
| **ARALIACEAE Juss.** |  |
| *Oreopanax*  *bogotensis* Cuatrec. | Tree |
| *Oreopanax*  *incisus* Decne. & Planch. | Tree |
| **ASTERACEAE Martinov** |  |
| *Ageratina*  *asclepiadea* (L.f.) R.M.King & H.Rob. | Shrub |
| *Ageratina*  *fastigiata* (Kunth) R.M.King & H.Rob*.* | Shrub |
| *Ageratina*  *glyptophlebia* (B.L.Rob.) R.M.King & H.Rob. | Shrub |
| *Baccharis*  *macrantha* Kunth | Shrub |
| *Barnadesia*  *spinosa* L.f. | Shrub |
| *Critoniopsis*  *bogotana* (Cuatrec.) H.Rob. | Tree |
| *Diplostephium rosmarinifolium* Wedd*.* | Tree |
| *Verbesina*  *arborea* Kunth | Tree |
| **BETULACEAE Gray** |  |
| *Alnus*  *acuminata* Kunth | Tree |
| **BORAGINACEAE Juss.** |  |
| *Cordia*  *cylindristachya* (Ruiz & Pav.) Roem. & Schult. | Tree |
| **CARICACEAE Dumort.** |  |
| *Vasconcellea*  *pubescens* A.DC. | Tree |
| **CELASTRACEAE R.Br** |  |
| *Maytenus*  *laxiflora* Triana & Planch. | Tree |
| **CHLORANTHACEAE R.Br. ex Sims** |  |
| *Hedyosmum* SW. sp | Tree |
| **CLETHRACEAE Klotzsch** |  |
| *Clethra*  *fimbriata* Kunth | Tree |
| *Clethra*  *lanata* M.Martens & Galeotti | Tree |
| **CLUSIACEAE Lindl.** |  |
| *Clusia*  *multiflora* Kunth | Tree |
| **CUNONIACEAE R.Br.** |  |
| *Weinmannia*  *tomentosa* L.f. | Tree |
| **ELAEOCARPACEAE Juss.** |  |
| *Vallea*  *stipularis* L.f. | Tree |
| **ERICACEAE Durande** |  |
| *Bejaria*  *resinosa* L.f. | Shrub |
| *Cavendishia*  *bracteata* (Ruiz & Pav. ex J.St.-Hil.) Hoerold | Shrub |
| *Cavendishia*  *nitida* | Shrub |
| *Macleania*  *rupestris* A.C.Sm. | Shrub |
| **ESCALLONIACEAE R.Br. ex Dumort.** |  |
| *Escallonia*  *discolor* Vent | Tree |
| **EUPHORBIACEAE Juss.** |  |
| *Croton*  *bogotanus* Cuatrec. | Tree |
| **FABACEAE Lindl.** |  |
| *Ulex*  *europaeus* L*.* | Shrub |
| **GENTIANACEAE Juss.** |  |
| *Macrocarpaea*  *glabra* Gilg | Shurb |
| **LAURACEAE Juss.** |  |
| *Aiouea*  *dubia* Mez | Tree |
| *Ocotea*  *calophylla* Mez | Tree |
| **LORANTHACEAE Juss.** |  |
| *Gaiadendron*  *punctatum* G.Don | Tree |
| **MELASTOMATACEAE Juss.** |  |
| *Bucquetia*  *glutinosa* DC. | Shrub |
| *Miconia*  *elaeoides* Naudin | Tree |
| *Miconia*  *ligustrina* Triana | Shrub |
| *Miconia*  *squamulosa* Triana | Shrub |
| **MELIACEAE Juss.** |  |
| *Cedrela*  *montana* Turcz. | Tree |
| **MYRICACEAE Rich. ex Kunth** |  |
| *Morella*  *parvifolia* (Benth.) Parra-Os. | Shrub |
| *Morella*  *pubescens* (Humb. & Bonpl. ex Willd.) Wilbur | Shrub |
| **MYRTACEAE Juss.** |  |
| *Myrcianthes*  *leucoxyla* (Ortega) McVaugh | Tree |
| **PIPERACEAE** **Giseke** |  |
| *Piper*  *bogotense* C.DC*.* | Shrub |
| **PRIMULACEAE Batsch ex Borkh.** |  |
| *Cybianthus*  *iteoides* (Benth.) G.Agostini | Shrub |
| *Myrsine*  *coriacea* (Sw.) R.Br. | Tree |
| *Myrsine*  *dependens* (Ruiz & Pav.) Spreng. | Tree |
| *Myrsine*  *guianensis* (Aubl.) Kuntze | Tree |
| **RHAMANCEAE Juss.** |  |
| *Rhamnus goudotiana* Triana & Planch. | Tree |
| *Rhamnus sphaerosperma* Sw. | Tree |
| **ROSACEAE Juss.** |  |
| *Hesperomeles*  *goudotiana* Killip | Tree |
| *Prunus*  *buxifolia* Koehne | Tree |
| **RUBIACEAE Juss.** |  |
| *Palicourea*  *angustifolia* Kunth | Shrub |
| *Palicourea*  *demissa* Standl. | Shrub |
| *Palicourea*  *lineariflora* Wernham | Shrub |
| *Psychotria*  *boqueronensis* Wernham | Shrub |
| **SALICACEAE Mirb.** |  |
| *Abatia*  *parviflora* Ruiz & Pav. | Shrub |
| *Xylosma*  *spiculifera* Triana & Planch. | Tree |
| **SOLANACEAE Juss.** |  |
| *Sessea*  *corymbosa* Goudot | Shrub |
| **SYMPLOCACEAE Desf.** |  |
| *Symplocos* Tree  *theiformis* (L.f.) Oken | Tree |
| **THYMELAEACEAE Juss.** |  |
| *Daphnopsis*  *caracasana* Meisn. | Tree |
| **VERBENACEAE J.St.-Hil.** |  |
| *Citharexylum*  *sulcatum* Moldenke | Tree |
| *Duranta*  *mutisii* L.f. | Shrub |
| *Lippia*  *hirsuta* L.f. | Tree |
| **VIBURNACEAE Raf.** |  |
| *Viburnum*  *triphyllum* Benth. | Tree |
| **WINTERACEAE R.Br. ex Lindl.** |  |
| *Drimys*  *granadensis* L.f. | Tree |

**Table S2.** Descriptive statistics from the leaf anatomical traits of the 63 plant species studied. Abbreviations of anatomical traits are depicted in Fig. 1 and Table 1.

| **Species** | **Code** | **AdCT** | **AbCT** | **AdET** | **AbET** | **AdHT** | **PMT** | **SMT** | **%AS** | **VBA** | **VBD** | **LT** |
| --- | --- | --- | --- | --- | --- | --- | --- | --- | --- | --- | --- | --- |
| *Abatia parviflora* | Abpa | 2.85 ± 0.50 | 2.14 ± 0.52 | 15.25 ± 3.88 | 12.72 ± 3.41 | 0.00 | 74.12 ± 18.66 | 37.86 ± 8.36 | 4.93 ± 8.36 | 1196.09 ± 482.45 | 43.89 ± 9.00 | 153.03 ± 22.80 |
| *Ageratina asclepiadea* | Agas | 3.52 ± 0.49 | 4.27 ± 0.59 | 13.30 ± 2.76 | 10.72 ± 1.96 | 17.25 ± 4.58 | 116.94 ± 15.39 | 136.60 ± 33.05 | 9.24 ± 33.05 | 6557.34 ± 1720.64 | 105.89 ± 18.09 | 316.20 ± 41.61 |
| *Ageratina fastigiata* | Agfa | 3.61 ± 0.88 | 2.76 ± 0.48 | 16.46 ± 2.41 | 11.56 ± 2.06 | 19.31 ± 3.82 | 101.31 ± 16.61 | 113.10 ± 18.73 | 13.44 ± 18.73 | 4370.23 ± 1435.09 | 87.59 ± 17.98 | 272.14 ± 20.68 |
| *Ageratina glyptophlebia* | Aggl | 3.95 ± 0.89 | 2.55 ± 0.48 | 19.15 ± 3.44 | 12.38 ± 3.50 | 0.00 | 89.06 ± 17.34 | 136.95 ± 19.45 | 32.48 ± 19.45 | 5001.21 ± 1523.43 | 98.41 ± 19.58 | 267.42 ± 14.56 |
| *Aiouea dubia* | Aidu | 15.91 ± 2.13 | 6.46 ± 1.04 | 20.81 ± 2.15 | 13.03 ± 1.80 | 0.00 | 122.86 ± 8.81 | 144.81 ± 14.33 | 16.05 ± 14.33 | 19932.30 ± 9910.47 | 177.87 ± 28.04 | 331.49 ± 28.43 |
| *Alnus acuminata* | Alac | 4.21 ± 0.84 | 2.78 ± 0.45 | 8.70 ± 1.45 | 10.92 ± 1.91 | 17.54 ± 5.65 | 98.51 ± 8.58 | 63.37 ± 8.24 | 4.81 ± 8.24 | 2479.95 ± 988.28 | 66.40 ± 13.05 | 206.90 ± 12.84 |
| *Baccharis macrantha* | Bama | 3.75 ± 0.60 | 4.40 ± 0.69 | 26.22 ± 3.63 | 24.48 ± 3.96 | 0.00 | 101.50 ± 13.65 | 127.26 ± 12.10 | 6.28 ± 12.10 | 7524.80 ± 4656.17 | 101.04 ± 29.58 | 311.05 ± 7.96 |
| *Barnadesia spinosa* | Basp | 2.97 ± 0.62 | 2.71 ± 0.75 | 18.49 ± 4.60 | 11.23 ± 1.91 | 0.00 | 110.87 ± 15.54 | 94.67 ± 17.25 | 18.80 ± 17.25 | 4780.09 ± 1954.92 | 91.39 ± 26.04 | 247.31 ± 15.20 |
| *Bejaria resinosa* | Bere | 11.27 ± 1.95 | 9.48 ± 1.55 | 30.77 ± 6.27 | 15.26 ± 3.48 | 61.49 ± 18.27 | 85.86 ± 20.29 | 169.85 ± 18.54 | 17.65 ± 18.54 | 17950.55 ± 9645.76 | 178.09 ± 60.28 | 391.27 ± 32.82 |
| *Bucquetia glutinosa* | Bugl | 3.27 ± 0.46 | 2.35 ± 0.45 | 24.60 ± 4.99 | 9.11 ± 2.39 | 0.00 | 77.78 ± 9.78 | 90.51 ± 17.26 | 5.81 ± 17.26 | 3378.14 ± 1153.89 | 75.92 ± 19.11 | 212.80 ± 14.34 |
| *Cavendishia bracteata* | Cabr | 10.25 ± 2.64 | 7.44 ± 1.54 | 23.75 ± 5.61 | 13.77 ± 1.97 | 32.55 ± 7.47 | 53.88 ± 7.47 | 176.49 ± 28.03 | 11.70 ± 28.03 | 19537.68 ± 13947.34 | 200.88 ± 81.40 | 332.97 ± 38.88 |
| *Cavendishia nitida* | Cani | 14.40 ± 1.12 | 10.74 ± 2.43 | 34.58 ± 6.41 | 17.00 ± 2.53 | 48.06 ± 9.08 | 147.78 ± 52.00 | 382.69 ± 29.22 | 11.52 ± 29.22 | 31378.98 ± 14104.09 | 235.41 ± 65.13 | 670.93 ± 37.73 |
| *Cedrela montana* | Cemo | 4.59 ± 1.22 | 3.20 ± 0.57 | 10.12 ± 3.50 | 7.38 ± 1.40 | 0.00 | 99.21 ± 10.67 | 105.58 ± 18.72 | 19.08 ± 18.72 | 5938.36 ± 1951.74 | 89.45 ± 14.93 | 235.82 ± 19.37 |
| *Citharexylum sulcatum* | Cisu | 7.84 ± 1.26 | 5.10 ± 0.83 | 10.72 ± 1.48 | 10.85 ± 1.89 | 35.31 ± 17.42 | 54.13 ± 11.68 | 111.33 ± 19.41 | 1.86 ± 19.41 | 11546.68 ± 4376.87 | 148.27 ± 28.14 | 232.55 ± 14.59 |
| *Clethra fimbriata* | Clfi | 8.54 ± 1.13 | 4.26 ± 1.24 | 34.93 ± 5.26 | 11.46 ± 1.82 | 92.26 ± 16.77 | 99.45 ± 19.61 | 105.89 ± 28.88 | 2.01 ± 28.88 | 17685.81 ± 13461.52 | 156.42 ± 62.60 | 362.91 ± 29.82 |
| *Clethra lanata* | Clla | 6.77 ± 1.28 | 4.38 ± 0.91 | 14.39 ± 4.98 | 7.50 ± 1.21 | 18.23 ± 6.94 | 57.51 ± 19.62 | 86.73 ± 36.26 | 5.96 ± 36.26 | 8358.35 ± 3049.15 | 135.35 ± 34.45 | 195.01 ± 71.10 |
| *Clusia multiflora* | Clmu | 10.70 ± 1.51 | 6.89 ± 1.75 | 13.46 ± 2.36 | 15.18 ± 6.18 | 111.73 ± 12.95 | 115.49 ± 40.60 | 567.48 ± 72.26 | 9.83 ± 72.26 | 59821.36 ± 23937.55 | 331.40 ± 89.50 | 832.06 ± 106.02 |
| *Critoniopsis bogotana* | Cribo | 2.42 ± 0.34 | 2.01 ± 0.30 | 10.94 ± 1.73 | 8.64 ± 1.76 | 0.00 | 53.06 ± 6.11 | 26.11 ± 5.00 | 2.69 ± 5.00 | 1431.78 ± 822.95 | 50.51 ± 18.96 | 105.23 ± 10.98 |
| *Croton bogotanus* | Crobo | 2.68 ± 0.46 | 2.30 ± 0.45 | 10.21 ± 1.58 | 9.09 ± 2.87 | 23.99 ± 10.73 | 72.95 ± 13.47 | 56.90 ± 11.72 | 7.58 ± 11.72 | 1756.91 ± 947.17 | 49.68 ± 14.19 | 157.62 ± 17.35 |
| *Cybianthus iteoides* | Cyit | 12.12 ± 2.37 | 7.98 ± 1.48 | 27.57 ± 3.93 | 21.52 ± 4.02 | 0.00 | 116.14 ± 40.00 | 352.03 ± 59.42 | 19.24 ± 59.42 | 28170.49 ± 19132.44 | 222.85 ± 81.32 | 566.15 ± 44.67 |
| *Daphnopsis caracasana* | Daca | 4.74 ± 1.07 | 4.72 ± 1.02 | 38.19 ± 5.17 | 22.58 ± 3.44 | 0.00 | 122.73 ± 23.64 | 247.38 ± 36.48 | 16.19 ± 36.48 | 10784.26 ± 4701.47 | 124.78 ± 30.22 | 449.25 ± 62.40 |
| *Diplostephium rosmarinifolium* | Diro | 12.24 ± 1.96 | 4.62 ± 0.64 | 32.10 ± 6.09 | 9.02 ± 2.39 | 0.00 | 45.59 ± 8.58 | 53.60 ± 18.17 | 4.16 ± 18.17 | 3979.29 ± 1312.25 | 77.10 ± 18.33 | 170.72 ± 18.75 |
| *Drymis granadiensis* | Drgr | 10.10 ± 1.54 | 10.44 ± 1.86 | 16.99 ± 2.88 | 18.02 ± 5.06 | 18.01 ± 4.03 | 53.79 ± 21.31 | 96.38 ± 22.18 | 14.71 ± 22.18 | 7038.63 ± 2679.94 | 108.98 ± 26.25 | 226.73 ± 31.55 |
| *Duranta mutisii* | Dumu | 7.10 ± 2.20 | 4.71 ± 0.97 | 10.02 ± 2.80 | 11.81 ± 3.36 | 17.65 ± 2.91 | 119.48 ± 9.91 | 123.21 ± 17.24 | 2.53 ± 17.24 | 4567.48 ± 1361.17 | 84.37 ± 14.46 | 307.47 ± 26.41 |
| *Escallonia discolor* | Esdi | 3.87 ± 0.80 | 2.73 ± 0.79 | 28.93 ± 4.20 | 9.20 ± 2.03 | 0.00 | 53.75 ± 16.88 | 95.52 ± 29.77 | 10.86 ± 29.77 | 6168.38 ± 3014.28 | 97.78 ± 29.10 | 195.96 ± 45.20 |
| *Gaiadendron punctatum* | Gapu | 10.93 ± 1.78 | 7.14 ± 2.00 | 22.17 ± 4.18 | 18.27 ± 5.23 | 0.00 | 117.95 ± 33.83 | 457.92 ± 140.37 | 12.46 ± 140.37 | 23958.70 ± 13312.66 | 220.39 ± 82.30 | 662.45 ± 190.87 |
| *Hesperomeles goudotiana* | Hego | 7.65 ±  1.27 | 5.52 ±  0.82 | 23.08 ± 5.86 | 10.74 ± 2.65 | 0.00 | 64.56 ±  10.39 | 115.98 ± 12.56 | 15.35 ± 12.56 | 17147.29 ± 7957.98 | 168.25 ± 38.30 | 233.26 ± 22.14 |
| *Hedyosmum* sp | Hesp | 4.08 ± 0.45 | 4.01 ± 0.70 | 28.26 ± 4.10 | 14.61 ± 2.29 | 0.00 | 152.55 ± 12.66 | 153.53 ± 13.78 | 6.52 ± 13.78 | 4791.12 ± 1564.56 | 87.64 ± 11.80 | 367.43 ± 8.40 |
| *Ilex kunthiana* | Ilku | 22.52 ± 3.55 | 13.14 ± 2.25 | 43.77 ± 11.03 | 18.06 ± 3.10 | 40.84 ± 10.87 | 174.97 ± 41.85 | 209.47 ± 25.28 | 12.21 ± 25.28 | 22663.36 ± 11273.61 | 191.64 ± 56.68 | 529.73 ± 24.16 |
| *Lippia hirsuta* | Lihi | 2.67 ± 0.60 | 1.89 ± 0.25 | 17.37 ± 3.34 | 8.44 ± 1.74 | 0.00 | 38.35 ± 5.72 | 36.42 ± 8.03 | 9.00 ± 8.03 | 973.91 ± 371.18 | 37.46 ± 8.05 | 102.82 ± 8.89 |
| *Macrocarpaea glabra* | Magl | 2.92 ± 0.39 | 2.17 ± 0.40 | 15.04 ± 2.47 | 11.21 ± 1.86 | 20.27 ± 4.79 | 155.34 ± 20.96 | 210.41 ± 21.40 | 13.09 ± 21.40 | 5284.07 ± 1246.07 | 93.52 ± 15.27 | 419.81 ± 10.04 |
| *Maytenus laxiflora* | Mala | 9.04 ± 1.41 | 8.65 ± 1.46 | 21.79 ± 4.00 | 21.22 ± 2.80 | 116.13 ± 12.31 | 154.52 ± 42.26 | 243.63 ± 39.91 | 4.65 ± 39.91 | 19949.28 ± 8921.23 | 181.13 ± 42.80 | 573.31 ± 63.98 |
| *Macleania rupestris* | Maru | 14.39 ± 2.37 | 9.94 ± 2.89 | 26.01 ± 4.87 | 18.02 ± 2.54 | 55.52 ± 26.92 | 131.24 ± 33.24 | 424.24 ± 55.14 | 18.40 ± 55.14 | 47403.07 ± 16308.24 | 271.20 ± 48.98 | 691.10 ± 34.32 |
| *Miconia elaeoides* | Miel | 4.11 ± 0.49 | 3.84 ± 0.70 | 8.79 ± 1.54 | 8.78 ± 1.34 | 47.21 ± 4.53 | 95.83 ± 17.69 | 113.16 ± 21.13 | 3.14 ± 21.13 | 4814.74 ± 1639.78 | 85.89 ± 16.66 | 296.90 ± 37.81 |
| *Miconia ligustrina* | Mili | 4.72 ± 0.62 | 3.13 ± 0.65 | 8.24 ± 3.04 | 7.99 ± 2.15 | 18.47 ± 3.24 | 89.09 ± 11.61 | 160.90 ± 27.47 | 7.45 ± 27.47 | 7686.99 ± 3961.06 | 110.98 ± 28.17 | 298.55 ± 34.78 |
| *Miconia squamulosa* | Misq | 5.87 ± 1.79 | 5.68 ± 1.49 | 13.50 ± 1.71 | 16.71 ± 4.60 | 24.72 ± 7.09 | 96.32 ± 10.32 | 129.00 ± 41.07 | 4.67 ± 41.07 | 2844.96 ± 999.01 | 64.69 ± 12.69 | 304.20 ± 48.64 |
| *Morella parvifolia* | Mopa | 7.15 ± 1.88 | 4.20 ± 0.61 | 12.55 ± 2.20 | 9.78 ± 1.69 | 0.00 | 84.01 ± 15.78 | 97.77 ± 17.70 | 26.13 ± 17.70 | 8343.10 ± 4227.85 | 116.93 ± 40.30 | 217.51 ± 34.93 |
| *Morella pubescens* | Mopu | 6.07 ± 2.22 | 3.67 ± 0.99 | 14.84 ± 1.96 | 10.70 ± 2.02 | 0.00 | 95.68 ± 12.82 | 124.26 ± 25.63 | 18.37 ± 25.63 | 8144.73 ± 3359.85 | 117.45 ± 28.64 | 253.18 ± 43.74 |
| *Myrsine coriacea* | Myco | 9.49 ± 2.87 | 5.08 ± 1.15 | 16.08 ± 3.21 | 13.66 ± 2.64 | 0.00 | 70.65 ± 16.54 | 146.70 ± 55.03 | 16.72 ± 55.03 | 13086.38 ± 9328.39 | 146.45 ± 69.39 | 262.03 ± 61.54 |
| *Myrsine dependens* | Myde | 6.65 ± 1.83 | 5.37 ± 1.08 | 22.22 ± 4.31 | 14.78 ± 2.25 | 0.00 | 68.54 ± 9.96 | 142.30 ± 17.34 | 10.96 ± 17.34 | 6641.82 ± 3013.32 | 101.80 ± 26.53 | 264.60 ± 11.84 |
| *Myrsine guianensis* | Mygu | 14.93 ± 2.57 | 11.14 ± 1.94 | 26.45 ± 2.89 | 19.42 ± 3.48 | 0.00 | 175.35 ± 23.73 | 290.48 ± 50.77 | 11.08 ± 50.77 | 20704.75 ± 8631.25 | 183.63 ± 43.20 | 547.21 ± 74.61 |
| *Myrcianthes leucoxyla* | Myle | 6.73 ± 1.35 | 5.79 ± 1.34 | 13.29 ± 2.38 | 10.95 ± 1.59 | 19.36 ± 3.25 | 122.19 ± 10.67 | 246.19 ± 33.17 | 12.25 ± 33.17 | 27895.39 ± 11253.46 | 205.04 ± 54.44 | 436.54 ± 31.66 |
| *Ocotea calophylla* | Occa | 9.43 ± 1.63 | 7.11 ± 1.64 | 19.88 ± 3.24 | 13.55 ± 2.08 | 32.11 ± 7.25 | 144.04 ± 9.65 | 176.98 ± 23.36 | 11.02 ± 23.36 | 28759.53 ± 19811.95 | 223.61 ± 64.57 | 402.67 ± 12.69 |
| *Oreopanax bogotensis* | Orbo | 16.09 ± 3.59 | 7.35 ± 1.58 | 26.32 ± 6.48 | 13.77 ± 2.61 | 57.42 ± 27.48 | 142.59 ± 39.11 | 209.70 ± 46.62 | 17.96 ± 46.62 | 17971.25 ± 9957.12 | 178.43 ± 50.57 | 488.21 ± 116.04 |
| *Oreopanax incisus* | Orin | 9.51 ± 1.81 | 5.69 ± 1.62 | 18.97 ± 3.36 | 8.25 ± 2.33 | 22.74 ± 4.82 | 77.79 ± 30.80 | 106.55 ± 37.77 | 14.73 ± 37.77 | 14283.58 ± 5032.75 | 167.31 ± 32.37 | 256.83 ± 77.58 |
| *Palicourea angustifolia* | Paan | 2.98 ± 0.35 | 2.49 ± 0.65 | 20.06 ± 1.87 | 10.31 ± 1.73 | 0.00 | 51.99 ± 10.65 | 41.25 ± 8.50 | 7.83 ± 8.50 | 1510.95 ± 941.25 | 45.80 ± 13.17 | 130.15 ± 19.79 |
| *Palicourea demissa* | Pade | 4.19 ± 0.70 | 3.39 ± 0.58 | 26.53 ± 9.28 | 14.03 ± 3.19 | 32.80 ± 7.55 | 121.36 ± 14.09 | 134.60 ± 15.06 | 3.45 ± 15.06 | 9820.00 ± 5365.91 | 118.85 ± 33.10 | 336.34 ± 29.15 |
| *Palicourea lineariflora* | Pali | 2.88 ± 0.48 | 1.94 ± 0.53 | 21.76 ± 3.66 | 11.96 ± 3.20 | 0.00 | 95.90 ± 18.54 | 78.00 ± 14.51 | 4.96 ± 14.51 | 5263.24 ± 3105.07 | 83.91 ± 22.20 | 224.05 ± 11.25 |
| *Piper bogotense* | Pibo | 3.54 ± 0.56 | 2.37 ± 0.43 | 10.94 ± 2.45 | 10.79 ± 2.86 | 39.30 ± 6.65 | 73.97 ± 9.93 | 66.42 ± 13.04 | 3.36 ± 13.04 | 1287.80 ± 886.30 | 41.99 ± 13.96 | 206.60 ± 10.06 |
| *Prunus buxifolia* | Prbu | 6.99 ± 1.18 | 4.99 ± 0.91 | 44.11 ± 15.12 | 17.78 ± 2.50 | 0.00 | 92.57 ± 10.58 | 174.65 ± 33.54 | 18.41 ± 33.54 | 17480.52 ± 11238.48 | 161.31 ± 64.02 | 340.63 ± 55.09 |
| *Psychotria boqueronensis* | Psbo | 5.22 ± 1.15 | 4.63 ± 1.23 | 29.53 ± 5.21 | 15.60 ± 2.48 | 0.00 | 92.22 ± 18.84 | 128.81 ± 17.66 | 9.89 ± 17.66 | 7461.37 ± 3120.99 | 105.87 ± 24.11 | 284.51 ± 25.62 |
| *Rhamnus goudotiana* | Rhgo | 2.55 ± 0.55 | 1.99 ± 0.39 | 13.96 ± 3.74 | 8.77 ± 2.03 | 0.00 | 47.15 ± 13.15 | 35.25 ± 6.01 | 10.76 ± 6.01 | 1741.06 ± 828.20 | 50.92 ± 12.85 | 118.14 ± 18.07 |
| *Rhamnus sphaerosperma* | Rhsp | 3.13 ± 0.52 | 2.29 ± 0.37 | 19.85 ± 4.99 | 11.30 ± 2.65 | 0.00 | 46.43 ± 7.99 | 41.53 ± 15.03 | 9.30 ± 15.03 | 2594.08 ± 1098.33 | 61.68 ± 15.47 | 138.72 ± 24.37 |
| *Sessea corymbosa* | Seco | 3.99 ± 0.73 | 3.50 ± 0.88 | 21.07 ± 2.44 | 15.42 ± 2.29 | 0.00 | 83.89 ± 13.21 | 184.33 ± 36.19 | 20.50 ± 36.19 | 5707.16 ± 2332.13 | 97.46 ± 23.15 | 316.02 ± 40.63 |
| *Symplocos theiformis* | Syth | 14.92 ± 2.10 | 7.46 ± 1.03 | 28.99 ± 2.67 | 10.35 ± 1.79 | 0.00 | 193.03 ± 16.81 | 235.56 ± 20.94 | 14.39 ± 20.94 | 8737.67 ± 2956.82 | 128.31 ± 22.20 | 485.86 ± 7.64 |
| *Ulex europaeus* | Uleu | 9.57 ± 1.46 | 4.39 ± 0.76 | 8.55 ± 1.64 | 8.52 ± 1.68 | 0.00 | 23.97 ± 6.68 | 38.87 ± 6.46 | 33.18 ± 6.46 | 22655.58 ± 15483.78 | 166.51 ± 37.15 | 105.70 ± 11.83 |
| *Varronia cylindristachya* | Vacy | 2.63 ± 0.57 | 1.65 ± 0.46 | 15.34 ± 4.16 | 6.47 ±  1.41 | 0.00 | 94.80 ±  10.49 | 57.39 ± 7.57 | 5.50 ± 7.57 | 2104.68 ± 474.67 | 56.85 ± 7.92 | 177.29 ± 16.22 |
| *Vasconcellea pubescens* | Vapu | 3.30 ±  0.72 | 2.61 ±  0.45 | 30.90 ± 7.73 | 14.14 ± 4.76 | 0.00 | 64.42 ± 14.84 | 58.09 ± 9.53 | 4.88 ± 9.53 | 1258.21 ± 763.86 | 43.78 ± 14.30 | 171.27 ± 27.78 |
| *Vallea stipularis* | Vast | 3.68 ± 0.50 | 2.50 ± 0.37 | 16.85 ± 4.89 | 14.19 ± 2.72 | 0.00 | 105.76 ± 17.08 | 84.36 ± 20.43 | 5.67 ± 20.43 | 4103.69 ± 1831.47 | 81.87 ± 20.54 | 232.93 ± 33.60 |
| *Verbesina arborea* | Vear | 3.49 ± 0.59 | 2.49 ± 0.52 | 14.14 ± 2.63 | 7.00 ± 1.42 | 0.00 | 56.23 ± 6.60 | 62.60 ± 5.94 | 7.63 ± 5.94 | 2370.40 ± 713.59 | 60.04 ± 10.37 | 153.20 ± 6.95 |
| *Viburnum triphyllum* | Vitr | 4.85 ± 0.92 | 3.40 ± 0.83 | 26.85 ± 5.50 | 14.06 ± 2.92 | 0.00 | 89.02 ± 12.57 | 93.98 ± 19.41 | 9.70 ± 19.41 | 4017.21 ± 1571.84 | 77.04 ±  15.91 | 228.00 ±  27.46 |
| *Weinmannia tomentosa* | Weto | 8.00 ± 2.91 | 3.99 ± 0.71 | 26.12 ± 4.18 | 8.65 ± 1.50 | 61.84 ± 10.88 | 86.64 ± 15.94 | 111.83 ± 18.45 | 4.74 ± 18.45 | 7690.44 ± 1842.61 | 110.11 ± 18.88 | 312.18 ± 29.56 |
| *Xylosma spiculifera* | Xysp | 13.00 ± 2.15 | 7.82 ± 1.67 | 14.82 ± 2.65 | 12.61 ± 2.09 | 15.67 ± 3.02 | 121.93 ± 18.93 | 247.35 ± 25.63 | 3.92 ±  25.63 | 29088.50 ± 11393.27 | 255.29 ± 37.67 | 453.47 ± 26.24 |
| Total |  | **7.10** ± 4.64 | **4.85** ± 2.87 | **20.63** ± 9.67 | **12.78** ± 4.88 | **17.74** ± 28.00 | **95.82** ± 41.27 | **148.77** ± 111.12 | **11.01** ± 111.12 | **11580.57** ± 13793.61 | **124.46** ± 74.27 | **313.88** ± 163.04 |

**Table S3.** Descriptive statistics from the foliar traits from the LES of the 63 plant species studied. Abbreviations of anatomical traits are depicted in Table 1.

| **Species** | **Code** | **LMA** | **LDMC** | **CN ratio** | **LCC area** | **LNC area** | **A max area** | **LCC mass** | **LNC mass** | **A max mass** |
| --- | --- | --- | --- | --- | --- | --- | --- | --- | --- | --- |
| *Abatia parviflora* | Abpa | 110.88 ± 14.66 | 413.44 ± 107.61 | 25.71 ± 4.46 | 54.55 ± 6.59 | 2.14 ± 0.16 | 5.33 ± 0.86 | 492.54 ± 13.65 | 19.56 ± 3.55 | 49.22 ± 13.05 |
| *Ageratina asclepiadea* | Agas | 110.67 ± 33.61 | 333.91 ± 33.42 | 29.75 ± 3.82 | 53.40 ± 15.97 | 1.78 ± 0.44 | 12.20 ± 5.26 | 482.65 ± 29.55 | 16.44 ± 2.65 | 118.07 ± 54.81 |
| *Ageratina fastigiata* | Agfa | 124.99 ± 24.30 | 338.88 ± 41.48 | 31.68 ± 6.67 | 67.03 ± 13.73 | 2.12 ± 0.10 | 9.83 ± 1.78 | 535.79 ± 10.18 | 17.35 ± 3.15 | 80.06 ± 18.02 |
| *Ageratina glyptophlebia* | Aggl | 107.35 ± 14.10 | 362.81 ± 100.23 | 33.95 ± 2.38 | 52.89 ± 10.54 | 1.55 ± 0.25 | 11.60 ± 2.96 | 490.10 ± 37.95 | 14.47 ± 1.39 | 107.54 ± 20.16 |
| *Aiouea dubia* | Aidu | 211.44 ± 24.00 | 474.42 ± 30.75 | 36.95 ± 4.39 | 130.04 ± 17.33 | 3.58 ± 0.24 | 6.14 ± 0.91 | 614.11 ± 18.81 | 17.03 ± 1.47 | 29.19 ± 1.38 |
| *Alnus acuminata* | Alac | 99.61 ± 17.63 | 364.19 ± 23.10 | 20.15 ± 3.54 | 53.67 ± 12.51 | 2.65 ± 0.23 | 11.23 ± 3.27 | 535.61 ± 31.64 | 26.98 ± 3.68 | 116.61 ± 46.21 |
| *Baccharis macrantha* | Bama | 121.21 ± 11.45 | 296.60 ± 7.46 | 35.08 ± 2.30 | 59.90 ± 9.00 | 1.72 ± 0.36 | 12.97 ± 0.68 | 493.12 ± 38.61 | 14.11 ± 1.68 | 107.76 ± 13.76 |
| *Barnadesia spinosa* | Basp | 53.74 ± 3.59 | 267.13 ± 34.83 | 17.66 ± 2.60 | 24.95 ± 2.30 | 1.43 ± 0.17 | 5.70 ± 0.69 | 464.09 ± 24.12 | 26.70 ± 4.60 | 106.32 ± 14.45 |
| *Bejaria resinosa* | Bere | 100.28 ± 31.75 | 372.69 ± 36.93 | 47.17 ± 3.61 | 50.24 ± 9.38 | 1.07 ± 0.25 | 1.53 ± 0.31 | 513.54 ± 61.52 | 10.88 ± 0.94 | 16.04 ± 4.94 |
| *Bucquetia glutinosa* | Bugl | 95.72 ± 21.91 | 351.50 ± 57.30 | 39.12 ± 2.73 | 44.07± 4.14 | 1.14 ± 0.19 | 13.32 ± 3.57 | 469.31 ± 58.74 | 11.97 ± 0.68 | 141.86 ± 43.32 |
| *Cavendishia bracteata* | Cabr | 213.26 ± 43.38 | 427.07 ± 25.03 | 62.57± 15.54 | 117.43 ± 25.83 | 1.94 ± 0.61 | 6.87 ± 1.67 | 549.64 ± 20.43 | 9.11 ± 1.98 | 32.69 ± 7.27 |
| *Cavendishia nitida* | Cani | 206.95 ± 18.70 | 344.40 ± 37.19 | 106.21 ± 2.10 | 107.02 ± 12.86 | 1.01 ± 0.11 | 5.73 ± 1.63 | 516.16 ± 17.40 | 4.86 ± 0.14 | 27.76 ± 7.35 |
| *Cedrela montana* | Cemo | 108.21 ± 39.16 | 499.88 ± 213.90 | 39.03 ± 4.17 | 51.77 ± 21.91 | 1.33 ± 0.54 | 4.50 ± 1.40 | 473.01 ± 46.34 | 12.15 ± 0.76 | 50.36 ± 36.42 |
| *Citharexylum sulcatum* | Cisu | 158.76 | 340.79 | 40.86 | 76.02 | 1.86 | 7.40 | 478.85 | 11.72 | 46.61 ± |
| *Clethra fimbriata* | Clfi | 226.75 ± 83.44 | 408.02± 130.20 | 54.18 ± 17.70 | 109.61 ± 41.49 | 2.00 ± 0.21 | 9.41 ± 1.38 | 481.61 ± 8.01 | 9.70 ± 3.71 | 44.96 ± 14.70 |
| *Clethra lanata* | Clla | 231.45 ± 4.74 | 391.37 ± 2.51 | 52.08 ± 0.06 | 125.43 ± 12.51 | 2.41 ± 0.24 | 6.01 ± 0.32 | 541.51 ± 42.96 | 10.40 ± 0.81 | 25.97 ± 1.91 |
| *Clusia multiflora* | Clmu | 246.60 ± 55.73 | 270.22 ± 65.49 | 69.31 ± 15.79 | 116.99 ± 28.31 | 1.70 ± 0.36 | 5.28 ± 1.92 | 473.24 ± 11.97 | 7.06 ± 1.41 | 21.35 ± 5.01 |
| *Critoniopsis bogotana* | Cribo | 95.83 ± 11.65 | 480.02 ± 65.41 | 26.93 ± 4.34 | 45.98 ± 5.55 | 1.76 ± 0.46 | 6.80 ± 1.97 | 479.90 ± 13.99 | 18.17 ± 3.32 | 70.22 ± 13.20 |
| *Croton bogotanus* | Crobo | 118.08 ± 10.95 | 314.95 ± 45.87 | 12.34 ± 1.12 | 56.26 ± 3.59 | 4.58 ± 0.48 | 14.07 ± 4.53 | 477.36 ± 16.55 | 38.85 ± 3.07 | 122.30 ± 51.68 |
| *Cybianthus iteoides* | Cyit | 92.01 | 221.35 | 28.46 | 50.48 | 1.77 | 6.00 | 548.72 | 19.28 | 65.21 |
| *Daphnopsis caracasana* | Daca | 114.48 ± 13.05 | 427.17 ± 137.73 | 27.34 ± 5.29 | 53.99 ± 6.96 | 2.04 ± 0.58 | 7.07 ± 2.24 | 471.80 ± 29.53 | 17.80 ± 4.20 | 60.74 ± 13.67 |
| *Diplostephium rosmarinifolium* | Diro | 164.29 ± 39.47 | 432.95 ± 86.74 | 31.16 ± 7.62 | 78.43 ± 18.64 | 2.74 ± 1.40 | 2.85 ± 1.78 | 477.86 ± 21.53 | 15.99 ± 4.14 | 16.54 ± 7.84 |
| *Drymis granadiensis* | Drgr | 135.87 ± 52.67 | 313.24 ± 96.45 | 39.98 ± 10.60 | 78.97 ± 32.74 | 1.89 ± 0.44 | 7.11 ± 2.33 | 572.84 ± 29.84 | 15.21 ± 4.45 | 62.16 ± 35.98 |
| *Duranta mutisii* | Dumu | 86.95 ± 19.17 | 449.48 ± 10.49 | 28.83 ± 4.96 | 40.49 ± 11.23 | 1.40 ± 0.26 | 7.93 ± 2.11 | 462.37 ± 29.79 | 16.24 ± 1.81 | 91.00 ± 14.56 |
| *Escallonia discolor* | Esdi | 97.59 | 312.33 | 47.73 | 49.53 | 1.04 | 2.60 | 507.46 | 10.63 | 26.64 |
| *Gaiadendron punctatum* | Gapu | 210.31 ± 33.38 | 433.02 ± 70.45 | 67.08 ± 14.05 | 120.59 ± 16.64 | 1.82 ± 0.19 | 7.63 ± 0.23 | 574.73 ± 12.82 | 8.89 ± 2.29 | 36.92 ± 5.93 |
| *Hesperomeles goudotiana* | Hego | 183.04 ± 31.71 | 515.42 ± 63.76 | 64.42 ± 8.93 | 92.60 ± 16.42 | 1.44 ± 0.25 | 8.17 ± 1.46 | 505.72 ± 20.23 | 7.96 ± 1.26 | 44.59 ± 0.44 |
| *Hedyosmum* sp | Hesp | 98.19 ± 13.30 | 229.92 ± 16.53 | 27.25 ± 1.85 | 42.48 ± 3.43 | 1.56 ± 0.16 | 6.27 ± 1.83 | 435.37 ± 36.70 | 16.09 ± 2.44 | 65.17 ± 24.49 |
| *Ilex kunthiana* | Ilku | 259.77 ± 55.28 | 561.65 ± 100.75 | 98.10 ± 19.81 | 146.53 ± 33.32 | 1.49 ± 0.10 | 1.87 ± 0.15 | 563.35 ± 23.74 | 5.88 ± 1.10 | 7.34 ± 1.13 |
| *Lippia hirsuta* | Lihi | 79.39 ± 3.19 | 389.79 ± 70.00 | 19.52 ± 3.66 | 38.47 ± 4.41 | 2.05 ± 0.63 | 10.57 ± 2.31 | 484.13 ± 43.91 | 25.59 ± 6.77 | 132.67 ± 24.94 |
| *Macrocarpaea glabra* | Magl | 75.65 ± 2.36 | 190.33 ± 4.59 | 17.42 ± 1.51 | 39.58 ± 2.55 | 2.30 ± 0.36 | 7.45 ± 2.56 | 522.89 ± 20.44 | 30.27 ± 3.87 | 98.05 ± 32.01 |
| *Maytenus laxiflora* | Mala | 230.75 ± 15.06 | 415.14 ± 6.41 | 54.21 ± 6.26 | 112.85 ± 5.02 | 2.10 ± 0.21 | 9.61 ± 0.27 | 490.48 ± 39.46 | 9.15 ± 1.47 | 41.81 ± 3.45 |
| *Macleania rupestris* | Maru | 210.71 ± 15.96 | 303.88 ± 10.72 | 107.34 ± 27.15 | 117.62 ± 6.36 | 1.16 ± 0.38 | 6.59 ± 0.53 | 559.57 ± 37.99 | 5.52 ± 1.86 | 31.51 ± 4.78 |
| *Miconia elaeoides* | Miel | 108.85 ± 16.47 | 299.18 ± 17.37 | 24.58 ± 4.84 | 49.21 ± 8.45 | 2.02 ± 0.32 | 8.58 ± 0.89 | 451.04 ± 10.30 | 18.78 ± 3.30 | 80.99 ± 21.53 |
| *Miconia ligustrina* | Mili | 151.55 ± 13.53 | 339.69 ± 23.48 | 33.27 ± 3.68 | 86.33 ± 19.72 | 2.65 ± 0.79 | 5.37 ± 0.75 | 564.79 ± 82.40 | 17.26 ± 3.97 | 35.30 ± 1.84 |
| *Miconia squamulosa* | Misq | 133.06 ± 19.71 | 377.19 ± 13.67 | 29.15 ± 4.05 | 58.69 ± 9.71 | 2.06 ± 0.53 | 6.00 ± 1.35 | 440.39 ± 13.39 | 15.30 ± 1.88 | 45.02 ± 8.28 |
| *Morella parvifolia* | Mopa | 126.24 ± 7.20 | 433.31 ± 10.46 | 30.49 ± 4.84 | 67.23 ± 1.87 | 2.24 ± 0.31 | 9.17 ± 0.32 | 533.30 ± 23.32 | 17.84 ± 3.28 | 72.86 ± 6.53 |
| *Morella pubescens* | Mopu | 144.21 ± 11.72 | 429.90 ± 31.87 | 27.78 ± 3.08 | 74.29 ± 11.25 | 2.67 ± 0.17 | 10.23 ± 2.46 | 513.34 ± 42.51 | 18.52 ± 0.55 | 72.20 ± 23.40 |
| *Myrsine coriacea* | Myco | 146.28 ± 38.70 | 445.40 ± 45.74 | 42.18 ± 11.37 | 81.87 ± 21.31 | 1.95 ± 0.30 | 6.45 ± 1.48 | 560.21 ± 2.99 | 13.92 ± 3.63 | 53.29 ± 28.68 |
| *Myrsine dependens* | Myde | 129.86 ± 13.66 | 444.12 ± 69.37 | 34.97 ± 5.09 | 65.27 ± 1.73 | 1.89 ± 0.29 | 6.02 ± 2.85 | 506.60 ± 58.09 | 14.83 ± 3.73 | 45.31 ± 18.35 |
| *Myrsine guianensis* | Mygu | 170.32 ± 38.59 | 383.49 ± 61.49 | 49.32± 10.63 | 91.15± 19.35 | 1.89 ± 0.34 | 9.02 ± 1.54 | 537.03 ± 18.50 | 11.57 ± 3.83 | 54.84 ± 14.70 |
| *Myrcianthes leucoxyla* | Myle | 189.29 ± 66.97 | 206.90 ± 47.27 | 50.03 ± 19.33 | 94.74 ± 32.12 | 1.96 ± 0.45 | 3.58 ± 0.95 | 502.53 ± 22.98 | 11.30 ± 5.08 | 19.93 ± 7.16 |
| *Ocotea calophylla* | Occa | 186.72 ± 26.14 | 345.77 ± 25.03 | 33.20 ± 2.95 | 106.26 ± 18.39 | 3.25 ± 0.86 | 7.86 ± 2.85 | 567.84 ± 29.37 | 17.24 ± 2.36 | 41.59 ± 12.79 |
| *Oreopanax bogotensis* | Orbo | 271.38 ± 79.06 | 396.94 ± 50.79 | 57.55 ± 17.15 | 131.43 ± 28.39 | 2.36 ± 0.49 | 8.03 ± 2.29 | 491.11 ± 35.19 | 9.32 ± 3.95 | 30.92 ± 12.07 |
| *Oreopanax incisus* | Orin | 214.16 ± 20.47 | 380.84 ± 27.76 | 44.28 ± 10.35 | 106.64 ± 10.50 | 2.50 ± 0.64 | 8.77 ± 2.71 | 498.48 ± 30.45 | 11.86 ± 3.88 | 41.47 ± 14.22 |
| *Palicourea angustifolia* | Paan | 79.89 ± 6.64 | 353.46 ± 14.00 | 26.73 ± 5.11 | 34.15 ± 5.05 | 1.33 ± 0.40 | 5.83 ± 1.39 | 426.33 ± 37.32 | 16.43 ± 3.93 | 72.52 ± 13.29 |
| *Palicourea demissa* | Pade | 93.58 ± 2.99 | 261.63 ± 24.83 | 18.53 ± 2.44 | 46.93 ± 1.08 | 2.56 ± 0.32 | 7.92 ± 0.30 | 501.65 ± 10.42 | 27.38 ± 3.52 | 84.71 ± 5.22 |
| *Palicourea lineariflora* | Pali | 68.30 ± 3.68 | 288.12 ± 40.20 | 16.23 ± 0.61 | 32.37 ± 2.60 | 2.00 ± 0.17 | 4.97 ± 2.61 | 473.95 ± 29.82 | 29.26 ± 2.56 | 74.25 ± 42.67 |
| *Piper bogotense* | Pibo | 61.27 ± 7.99 | 229.58 ± 5.87 | 13.70 ± 0.84 | 26.47 ± 1.54 | 1.94 ± 0.14 | 8.03 ± 1.19 | 435.61 ± 44.46 | 31.77 ± 2.11 | 132.80 ± 27.51 |
| *Prunus buxifolia* | Prbu | 194.61 ± 17.95 | 496.31 ± 20.34 | 46.53 ± 6.52 | 103.99 ± 10.89 | 2.24 ± 0.11 | 5.57 ± 2.10 | 534.08 ± 15.84 | 11.63 ± 1.67 | 28.55 ± 9.79 |
| *Psychotria boqueronensis* | Psbo | 92.20 ± 25.87 | 340.88 ± 70.66 | 26.93 ± 2.90 | 44.63 ± 14.35 | 1.63 ± 0.38 | 4.03 ± 0.71 | 479.40 ± 27.81 | 17.87 ± 1.02 | 45.08 ± 8.04 |
| *Rhamnus goudotiana* | Rhgo | 63.87 ± 4.91 | 351.65 ± 26.35 | 19.79 ± 0.73 | 28.38 ± 2.53 | 1.43 ± 0.12 | 8.67 ± 0.25 | 447.97 ± 70.76 | 22.61 ± 3.25 | 136.39 ± 13.80 |
| *Rhamnus sphaerosperma* | Rhsp | 87.44 ± 13.25 | 377.42 ± 28.11 | 28.76 ± 2.42 | 38.98 ± 5.30 | 1.37 ± 0.31 | 7.33 ± 2.02 | 446.53 ± 14.32 | 15.59 ± 1.24 | 82.97 ± 11.74 |
| *Sessea corymbosa* | Seco | 127.25 | 278.77 | 22.79 | 57.42 | 2.52 | 4.80 | 451.28 | 19.80 | 37.72 |
| *Symplocos theiformis* | Syth | 138.51 ± 7.08 | 189.70 ± 36.17 | 52.09 ± 7.31 | 65.72 ± 9.09 | 1.27 ± 0.15 | 7.35 ± 0.42 | 473.10 ± 42.34 | 9.15 ± 0.85 | 53.21 ± 4.64 |
| *Ulex europaeus* | Uleu | 184.71 ± 9.08 | 402.67 ± 28.86 | 42.18 ± 2.93 | 108.45 ± 8.97 | 2.57 ± 0.03 | 1.95 ± 0.64 | 586.66 ± 19.73 | 13.92 ± 0.50 | 10.49 ± 2.93 |
| *Varronia cylindristachya* | Vacy | 116.68 ± 17.31 | 321.91 ± 35.10 | 32.73 ± 1.37 | 47.63 ± 6.27 | 1.46 ± 0.24 | 13.77 ± 5.02 | 409.37 ± 30.37 | 12.54 ± 1.40 | 121.53 ± 57.05 |
| *Vasconcellea pubescens* | Vapu | 63.15 | 120.16 | 9.68 | 28.11 | 2.90 | 5.60 | 445.20 | 46.00 | 88.68 |
| *Vallea stipularis* | Vast | 77.56 ± 23.55 | 628.29 ± 169.80 | 32.08 ± 1.10 | 36.95± 12.09 | 1.16 ± 0.42 | 6.60 ± 2.38 | 473.58 ± 17.42 | 14.78 ± 0.98 | 84.86 ± 10.56 |
| *Verbesina arborea* | Vear | 62.68 ± 11.97 | 178.53 ± 23.76 | 12.67 ± 1.72 | 30.38 ± 6.62 | 2.39 ± 0.29 | 13.92 ± 3.37 | 482.98 ± 18.97 | 38.49 ± 4.04 | 220.73 ± 21.32 |
| *Viburnum triphyllum* | Vitr | 77.83 ± 9.72 | 306.06 ± 30.13 | 36.10 ± 2.31 | 40.53 ± 4.47 | 1.12 ± 0.07 | 5.47 ± 1.82 | 521.48 ± 14.46 | 14.49 ± 1.03 | 70.49 ± 21.32 |
| *Weinmannia tomentosa* | Weto | 127.84 ± 23.50 | 357.77 ± 16.19 | 37.11 ± 8.10 | 64.54 ± 9.25 | 1.76 ± 0.19 | 1.53 ± 0.23 | 507.54 ± 22.08 | 14.29 ± 4.20 | 12.11 ± 1.54 |
| *Xylosma spiculifera* | Xysp | 243.04 ± 100.65 | 512.24 ± 22.69 | 74.96 ± 9.95 | 127.78 ± 57.87 | 1.73 ± 0.88 | 7.88 ± 1.69 | 517.19 ± 39.44 | 6.96 ± 0.75 | 35.40 ± 10.61 |
| Total |  | **140.49 ± 64.25** | **365.68 ± 107.11** | **39.59 ± 22.08** | **71.61 ± 35.79** | **1.95 ± 0.72** | **7.43 ± 3.40** | **501.12 ± 50.39** | **16.21 ± 8.13** | **65.50 ± 44.23** |

**Table S4.** Abbreviations and units of the leaf functional traits analyzed. Anatomical traits include tissue thickness measurements, tissue proportions (expressed as percentages of total leaf thickness), tissue ratios, and cell shape descriptors. Foliar traits from the Leaf economic spectrum (LES) include traits per unit mass and per unit area.

| **Abbreviation** | **Leaf functional trait** | **Unit** |
| --- | --- | --- |
|  | **Thickness measurements** |  |
| AdCT | Adaxial cuticle thickness | µm |
| AbCT | Abaxial cuticle thickness | µm |
| AdET | Adaxial epidermis thickness | µm |
| AbET | Abaxial epidermis thickness | µm |
| AdHT | Adaxial hypodermis thickness | µm |
| PMT | Palisade mesophyll thickness | µm |
| SMT | Spongy mesophyll thickness | µm |
| VBD | Vascular bundle diameter | µm |
| LT | Leaf thickness | µm |
| AS | Air space | µm^2^ |
| VBA | Vascular bundle area | µm^2^ |
| LTA | Leaf total area | µm^2^ |
| LACT | Leaf area cell tissue | µm^2^ |
|  | **Percentage of tissues (relative to LT)** |  |
| %AdCT | Percentage of Adaxial cuticle thickness | % |
| %AbCT | Percentage of Abaxial cuticle thickness | % |
| %AdET | Percentage of Adaxial epidermis thickness | % |
| %AbET | Percentage of Abaxial epidermis thickness | % |
| %AdHT | Percentage of Adaxial hypodermis thickness | % |
| %PMT | Percentage of Palisade mesophyll thickness | % |
| %SMT | Percentage of Spongy mesophyll thickness | % |
| %AS | Percentage of Air space | % |
|  | **Tissue ratios** |  |
| AdET/PMT | Adaxial epidermis thickness / Palisade mesophyll thickness | Adimensional |
| AdET/SMT | Adaxial epidermis thickness/ Spongy mesophyll thickness | Adimensional |
| PMT/SMT | Palisade mesophyll thickness/ Spongy mesophyll thickness | Adimensional |
| AdET/AbET | Adaxial epidermis thickness/ Abaxial epidermis thickness | Adimensional |
| AdCT/AbCT | Adaxial cuticle thickness/ Abaxial cuticle thickness | Adimensional |
|  | **Cell shapes** |  |
| AdEw | Adaxial epidermis cell width | µm |
| AdEl | Adaxial epidermis cell long | µm |
| AdHw | Adaxial hypodermis cell width | µm |
| AdHl | Adaxial hypodermis cell long | µm |
| PMw | Palisade mesophyll cell width | µm |
| PMl | Palisade mesophyll cell long | µm |
| SMw | Spongy mesophyll cell width | µm |
| SMl | Spongy mesophyll cell long | µm |
| AbEw | Abaxial epidermis cell width | µm |
| AbEl | Abaxial epidermis cell long | µm |
| As AdEc | Aspect ratio of Adaxial epidermis cells | Adimensional |
| As PMc | Aspect ratio of Palisade mesophyll cells | Adimensional |
| As SMc | Aspect ratio of Spongy mesophyll cells | Adimensional |
| As AbEc | Aspect ratio of Abaxial epidermis cells | Adimensional |
|  | **Foliar trait from LES** |  |
| LMA | Leaf mass per area | g m^-2^ |
| LDMC | Leaf dry matter content | mg g^-1^ |
| C:N ratio | Carbon: Nitrogen ratio | Adimensional |
| LCC area | Leaf carbon content per unit area | g m^-2^ |
| LNC area | Leaf nitrogen content per unit area | g m^-2^ |
| A max area | Leaf photosynthetic capacity per unit area | umol m^-2^ s^-1^ |
| LCC mass | Leaf carbon content per unit mass | mg g^-1^ |
| LNC mass | Leaf nitrogen content per unit mass | mg g^-1^ |
| A max mass | Leaf photosynthetic capacity per unit mass | nmol g^-1^ s^-1^ |

**Table S5.** Correlation matrix between absolute anatomical measurements and foliar traits within the LES, showing the Spearman´s rho coefficient. * *P* < 0.05, ***P* < 0.01, ****P* < 0.001.

| **Trait** | | **LT** | | **AdCT** | | **AbCT** | | **AdHT** | | **AdET** | | **AbET** | | **PMT** | | **SMT** | | **VBD** | | **VBA** | | **AS** | | **LTA** | | **LACT** | | **LMA** | | **LDMC** | | **CN** | | **C area** | | **N area** | | **A max area** | | **C mass** | | **N mass** | | **A max mass** | |
| --- | --- | --- | --- | --- | --- | --- | --- | --- | --- | --- | --- | --- | --- | --- | --- | --- | --- | --- | --- | --- | --- | --- | --- | --- | --- | --- | --- | --- | --- | --- | --- | --- | --- | --- | --- | --- | --- | --- | --- | --- | --- | --- | --- | --- | --- |
| **LT** |  | — |  |  |  |  |  |  |  |  |  |  |  |  |  |  |  |  |  |  |  |  |  |  |  |  |  |  |  |  |  |  |  |  |  |  |  |  |  |  |  |  |  |  |  |
| **AdCT** |  | 0.650 | *** | — |  |  |  |  |  |  |  |  |  |  |  |  |  |  |  |  |  |  |  |  |  |  |  |  |  |  |  |  |  |  |  |  |  |  |  |  |  |  |  |  |  |
| **AbCT** |  | 0.739 | *** | 0.921 | *** | — |  |  |  |  |  |  |  |  |  |  |  |  |  |  |  |  |  |  |  |  |  |  |  |  |  |  |  |  |  |  |  |  |  |  |  |  |  |  |  |
| **AdHT** |  | 0.455 | *** | 0.358 | ** | 0.387 | ** | — |  |  |  |  |  |  |  |  |  |  |  |  |  |  |  |  |  |  |  |  |  |  |  |  |  |  |  |  |  |  |  |  |  |  |  |  |  |
| **AdET** |  | 0.391 | ** | 0.333 | ** | 0.346 | ** | -0.082 |  | — |  |  |  |  |  |  |  |  |  |  |  |  |  |  |  |  |  |  |  |  |  |  |  |  |  |  |  |  |  |  |  |  |  |  |  |
| **AbET** |  | 0.644 | *** | 0.402 | ** | 0.573 | *** | 0.107 |  | 0.544 | *** | — |  |  |  |  |  |  |  |  |  |  |  |  |  |  |  |  |  |  |  |  |  |  |  |  |  |  |  |  |  |  |  |  |  |
| **PMT** |  | 0.830 | *** | 0.380 | ** | 0.454 | *** | 0.299 | * | 0.230 |  | 0.488 | *** | — |  |  |  |  |  |  |  |  |  |  |  |  |  |  |  |  |  |  |  |  |  |  |  |  |  |  |  |  |  |  |  |
| **SMT** |  | 0.958 | *** | 0.650 | *** | 0.752 | *** | 0.344 | ** | 0.331 | ** | 0.630 | *** | 0.734 | *** | — |  |  |  |  |  |  |  |  |  |  |  |  |  |  |  |  |  |  |  |  |  |  |  |  |  |  |  |  |  |
| **VBD** |  | 0.780 | *** | 0.834 | *** | 0.849 | *** | 0.392 | ** | 0.281 | * | 0.429 | *** | 0.496 | *** | 0.801 | *** | — |  |  |  |  |  |  |  |  |  |  |  |  |  |  |  |  |  |  |  |  |  |  |  |  |  |  |  |
| **VBA** |  | 0.772 | *** | 0.830 | *** | 0.836 | *** | 0.382 | ** | 0.282 | * | 0.432 | *** | 0.501 | *** | 0.787 | *** | 0.992 | *** | — |  |  |  |  |  |  |  |  |  |  |  |  |  |  |  |  |  |  |  |  |  |  |  |  |  |
| **AS** |  | 0.684 | *** | 0.549 | *** | 0.593 | *** | 0.046 |  | 0.312 | * | 0.475 | *** | 0.504 | *** | 0.762 | *** | 0.694 | *** | 0.678 | *** | — |  |  |  |  |  |  |  |  |  |  |  |  |  |  |  |  |  |  |  |  |  |  |  |
| **LTA** |  | 0.995 | *** | 0.654 | *** | 0.737 | *** | 0.463 | *** | 0.391 | ** | 0.629 | *** | 0.823 | *** | 0.950 | *** | 0.777 | *** | 0.770 | *** | 0.679 | *** | — |  |  |  |  |  |  |  |  |  |  |  |  |  |  |  |  |  |  |  |  |  |
| **LACT** |  | 0.974 | *** | 0.629 | *** | 0.718 | *** | 0.514 | *** | 0.366 | ** | 0.611 | *** | 0.822 | *** | 0.909 | *** | 0.738 | *** | 0.738 | *** | 0.559 | *** | 0.980 | *** | — |  |  |  |  |  |  |  |  |  |  |  |  |  |  |  |  |  |  |  |
| **LMA** |  | 0.519 | *** | 0.753 | *** | 0.718 | *** | 0.429 | *** | 0.122 |  | 0.201 |  | 0.274 | * | 0.533 | *** | 0.765 | *** | 0.753 | *** | 0.404 | ** | 0.517 | *** | 0.491 | *** | — |  |  |  |  |  |  |  |  |  |  |  |  |  |  |  |  |  |
| **LDMC** |  | -0.040 |  | 0.298 | * | 0.207 |  | -0.122 |  | -0.006 |  | 0.019 |  | -0.093 |  | -0.026 |  | 0.184 |  | 0.177 |  | 0.024 |  | -0.037 |  | -0.068 |  | 0.399 | ** | — |  |  |  |  |  |  |  |  |  |  |  |  |  |  |  |
| **CN** |  | 0.533 | *** | 0.756 | *** | 0.731 | *** | 0.326 | ** | 0.280 | * | 0.251 | * | 0.263 | * | 0.540 | *** | 0.775 | *** | 0.761 | *** | 0.475 | *** | 0.531 | *** | 0.518 | *** | 0.803 | *** | 0.371 | ** | — |  |  |  |  |  |  |  |  |  |  |  |  |  |
| **C area** |  | 0.529 | *** | 0.785 | *** | 0.740 | *** | 0.401 | ** | 0.117 |  | 0.241 |  | 0.287 | * | 0.553 | *** | 0.799 | *** | 0.788 | *** | 0.460 | *** | 0.526 | *** | 0.490 | *** | 0.982 | *** | 0.408 | ** | 0.799 | *** | — |  |  |  |  |  |  |  |  |  |  |  |
| **N area** |  | -0.104 |  | 0.012 |  | -0.061 |  | 0.107 |  | -0.230 |  | -0.133 |  | -0.074 |  | -0.074 |  | -0.016 |  | -0.007 |  | -0.125 |  | -0.109 |  | -0.133 |  | 0.228 |  | 0.012 |  | -0.312 | * | 0.253 | * | — |  |  |  |  |  |  |  |  |  |
| **A max area** |  | -0.100 |  | -0.248 |  | -0.199 |  | 0.019 |  | -0.258 | * | -0.183 |  | 0.070 |  | -0.117 |  | -0.175 |  | -0.203 |  | -0.171 |  | -0.108 |  | -0.103 |  | -0.070 |  | -0.021 |  | -0.167 |  | -0.080 |  | 0.152 |  | — |  |  |  |  |  |  |  |
| **C mass** |  | 0.311 | * | 0.559 | *** | 0.496 | *** | 0.151 |  | 0.082 |  | 0.214 |  | 0.145 |  | 0.371 | ** | 0.618 | *** | 0.608 | *** | 0.441 | *** | 0.309 | * | 0.264 | * | 0.504 | *** | 0.260 | * | 0.481 | *** | 0.636 | *** | 0.245 |  | -0.116 |  | — |  |  |  |  |  |
| **N mass** |  | -0.488 | *** | -0.665 | *** | -0.659 | *** | -0.290 | * | -0.309 | * | -0.233 |  | -0.238 |  | -0.485 | *** | -0.678 | *** | -0.664 | *** | -0.399 | ** | -0.487 | *** | -0.486 | *** | -0.726 | *** | -0.355 | ** | -0.971 | *** | -0.691 | *** | 0.440 | *** | 0.161 |  | -0.299 | * | — |  |  |  |
| **A max mass** |  | -0.444 | *** | -0.726 | *** | -0.656 | *** | -0.329 | ** | -0.304 | * | -0.222 |  | -0.126 |  | -0.460 | *** | -0.689 | *** | -0.695 | *** | -0.401 | ** | -0.453 | *** | -0.428 | *** | -0.744 | *** | -0.312 | * | -0.705 | *** | -0.742 | *** | -0.048 |  | 0.666 | *** | -0.470 | *** | 0.645 | *** | — |  |

**Table S6.** Correlation matrix between percentage (%) of anatomical measurements and foliar traits within the LES, showing the Spearman´s rho coefficient. * *P* < 0.05, ***P* < 0.01, ****P* < 0.001.

| **Trait** | | **% AdCT** | | **% AbCT** | | **% AbET** | | **% AbHT** | | **% PMT** | | **% SMT** | | **% AS** | | **LMA** | | **LDMC** | | **CN** | | **C area** | | **N area** | | **A max area** | | **C mass** | | **N mass** | | **A max mass** | |
| --- | --- | --- | --- | --- | --- | --- | --- | --- | --- | --- | --- | --- | --- | --- | --- | --- | --- | --- | --- | --- | --- | --- | --- | --- | --- | --- | --- | --- | --- | --- | --- | --- | --- |
| **% AdCT** |  | — |  |  |  |  |  |  |  |  |  |  |  |  |  |  |  |  |  |  |  |  |  |  |  |  |  |  |  |  |  |  |  |
| **% AbCT** |  | 0.868 | *** | — |  |  |  |  |  |  |  |  |  |  |  |  |  |  |  |  |  |  |  |  |  |  |  |  |  |  |  |  |  |
| **% AbET** |  | 0.078 |  | 0.280 | * | — |  |  |  |  |  |  |  |  |  |  |  |  |  |  |  |  |  |  |  |  |  |  |  |  |  |  |  |
| **% AbHT** |  | 0.047 |  | -0.066 |  | -0.052 |  | — |  |  |  |  |  |  |  |  |  |  |  |  |  |  |  |  |  |  |  |  |  |  |  |  |  |
| **% PMT** |  | -0.260 | * | -0.252 | * | 0.306 | * | -0.014 |  | — |  |  |  |  |  |  |  |  |  |  |  |  |  |  |  |  |  |  |  |  |  |  |  |
| **% SMT** |  | -0.034 |  | -0.046 |  | -0.506 | *** | -0.107 |  | -0.604 | *** | — |  |  |  |  |  |  |  |  |  |  |  |  |  |  |  |  |  |  |  |  |  |
| **% AS** |  | 0.136 |  | 0.098 |  | -0.130 |  | -0.029 |  | -0.229 |  | 0.549 | *** | — |  |  |  |  |  |  |  |  |  |  |  |  |  |  |  |  |  |  |  |
| **LMA** |  | 0.437 | *** | 0.323 | * | -0.482 | *** | 1.580×10^-4^ |  | -0.585 | *** | 0.450 | *** | 0.187 |  | — |  |  |  |  |  |  |  |  |  |  |  |  |  |  |  |  |  |
| **LDMC** |  | 0.505 | *** | 0.441 | *** | 0.127 |  | -0.077 |  | -0.074 |  | -0.002 |  | 0.075 |  | 0.399 | ** | — |  |  |  |  |  |  |  |  |  |  |  |  |  |  |  |
| **CN** |  | 0.426 | *** | 0.298 | * | -0.502 | *** | -0.029 |  | -0.615 | *** | 0.507 | *** | 0.246 |  | 0.803 | *** | 0.371 | ** | — |  |  |  |  |  |  |  |  |  |  |  |  |  |
| **C area** |  | 0.460 | *** | 0.335 | ** | -0.466 | *** | -0.005 |  | -0.600 | *** | 0.493 | *** | 0.257 | * | 0.982 | *** | 0.408 | ** | 0.799 | *** | — |  |  |  |  |  |  |  |  |  |  |  |
| **N area** |  | 0.093 |  | 0.067 |  | 0.084 |  | 0.088 |  | 0.002 |  | -0.076 |  | -0.038 |  | 0.228 |  | 0.012 |  | -0.312 | * | 0.253 | * | — |  |  |  |  |  |  |  |  |  |
| **A max area** |  | -0.127 |  | -0.131 |  | -0.020 |  | 0.115 |  | 0.276 | * | -0.138 |  | -0.167 |  | -0.070 |  | -0.021 |  | -0.167 |  | -0.080 |  | 0.152 |  | — |  |  |  |  |  |  |  |
| **C mass** |  | 0.427 | *** | 0.314 | * | -0.225 |  | -0.146 |  | -0.410 | *** | 0.434 | *** | 0.402 | ** | 0.504 | *** | 0.260 | * | 0.481 | *** | 0.636 | *** | 0.245 |  | -0.116 |  | — |  |  |  |  |  |
| **N mass** |  | -0.357 | ** | -0.264 | * | 0.462 | *** | 0.039 |  | 0.558 | *** | -0.436 | *** | -0.165 |  | -0.726 | *** | -0.355 | ** | -0.971 | *** | -0.691 | *** | 0.440 | *** | 0.161 |  | -0.299 | * | — |  |  |  |
| **A max mass** |  | -0.439 | *** | -0.349 | ** | 0.366 | ** | 0.049 |  | 0.658 | *** | -0.413 | *** | -0.239 |  | -0.744 | *** | -0.312 | * | -0.705 | *** | -0.742 | *** | -0.048 |  | 0.666 | *** | -0.470 | *** | 0.645 | *** | — |  |

**Table S7.** Correlation matrix between anatomical ratios and foliar traits within the LES, showing the Spearman´s rho coefficient. * *P* < 0.05, ***P* < 0.01, ****P* < 0.001.

| **Trait** | | **AdET/PMT** | | **AdET/SMT** | | **PMT/SMT** | | **AdCT/AbCT** | | **AdET/ AbET** | | **LMA** | | **LDMC** | | **CN** | | **C area** | | **N area** | | **A max area** | | **C mass** | | **N mass** | | **A max mass** | |
| --- | --- | --- | --- | --- | --- | --- | --- | --- | --- | --- | --- | --- | --- | --- | --- | --- | --- | --- | --- | --- | --- | --- | --- | --- | --- | --- | --- | --- | --- |
| **AdET/PMT** |  | — |  |  |  |  |  |  |  |  |  |  |  |  |  |  |  |  |  |  |  |  |  |  |  |  |  |  |  |
| **AdET/SMT** |  | 0.683 | *** | — |  |  |  |  |  |  |  |  |  |  |  |  |  |  |  |  |  |  |  |  |  |  |  |  |  |
| **PMT/SMT** |  | -0.127 |  | 0.594 | *** | — |  |  |  |  |  |  |  |  |  |  |  |  |  |  |  |  |  |  |  |  |  |  |  |
| **AdCT/AbCT** |  | -0.004 |  | -0.044 |  | -0.091 |  | — |  |  |  |  |  |  |  |  |  |  |  |  |  |  |  |  |  |  |  |  |  |
| **AdET/ AbET** |  | 0.627 | *** | 0.566 | *** | 0.078 |  | 0.200 |  | — |  |  |  |  |  |  |  |  |  |  |  |  |  |  |  |  |  |  |  |
| **LMA** |  | -0.053 |  | -0.407 | ** | -0.575 | *** | 0.363 | ** | -0.041 |  | — |  |  |  |  |  |  |  |  |  |  |  |  |  |  |  |  |  |
| **LDMC** |  | 0.116 |  | 0.060 |  | -0.038 |  | 0.326 | ** | -0.017 |  | 0.399 | ** | — |  |  |  |  |  |  |  |  |  |  |  |  |  |  |  |
| **CN** |  | 0.096 |  | -0.340 | ** | -0.635 | *** | 0.365 | ** | 0.111 |  | 0.803 | *** | 0.371 | ** | — |  |  |  |  |  |  |  |  |  |  |  |  |  |
| **C area** |  | -0.054 |  | -0.443 | *** | -0.610 | *** | 0.384 | ** | -0.085 |  | 0.982 | *** | 0.408 | ** | 0.799 | *** | — |  |  |  |  |  |  |  |  |  |  |  |
| **N area** |  | -0.139 |  | -0.059 |  | 0.047 |  | 0.094 |  | -0.189 |  | 0.228 |  | 0.012 |  | -0.312 | * | 0.253 | * | — |  |  |  |  |  |  |  |  |  |
| **A max area** |  | -0.289 | * | -0.085 |  | 0.234 |  | -0.063 |  | -0.145 |  | -0.070 |  | -0.021 |  | -0.167 |  | -0.080 |  | 0.152 |  | — |  |  |  |  |  |  |  |
| **C mass** |  | 0.045 |  | -0.320 | * | -0.512 | *** | 0.282 | * | -0.097 |  | 0.504 | *** | 0.260 | * | 0.481 | *** | 0.636 | *** | 0.245 |  | -0.116 |  | — |  |  |  |  |  |
| **N mass** |  | -0.128 |  | 0.261 | * | 0.559 | *** | -0.311 | * | -0.171 |  | -0.726 | *** | -0.355 | ** | -0.971 | *** | -0.691 | *** | 0.440 | *** | 0.161 |  | -0.299 | * | — |  |  |  |
| **A max mass** |  | -0.206 |  | 0.215 |  | 0.604 | *** | -0.308 | * | -0.145 |  | -0.744 | *** | -0.312 | * | -0.705 | *** | -0.742 | *** | -0.048 |  | 0.666 | *** | -0.470 | *** | 0.645 | *** | — |  |

**Table S8.** Correlation matrix between cell shape traits and foliar traits within the LES, showing Spearman’s rho coefficients.* *P* < 0.05, ***P* < 0.01, ****P* < 0.001.

| **Trait** | | **As AdEc** | | **As AbEc** | | **As AdHc** | | **As PMc** | | **As SMc** | | **AdEw** | | **AdEl** | | **AdHw** | | **AdHl** | | **PMw** | | **PMl** | | **SMw** | | **SMl** | | **AbEw** | | **AbEl** | | **LMA** | | **LDMC** | | **CN** | | **C area** | | **N area** | | **A max area** | | **C mass** | | **N mass** | | **A max mass** | |
| --- | --- | --- | --- | --- | --- | --- | --- | --- | --- | --- | --- | --- | --- | --- | --- | --- | --- | --- | --- | --- | --- | --- | --- | --- | --- | --- | --- | --- | --- | --- | --- | --- | --- | --- | --- | --- | --- | --- | --- | --- | --- | --- | --- | --- | --- | --- | --- | --- | --- |
| **As AdEc** |  | — |  |  |  |  |  |  |  |  |  |  |  |  |  |  |  |  |  |  |  |  |  |  |  |  |  |  |  |  |  |  |  |  |  |  |  |  |  |  |  |  |  |  |  |  |  |  |  |
| **As AbEc** |  | -0.114 |  | — |  |  |  |  |  |  |  |  |  |  |  |  |  |  |  |  |  |  |  |  |  |  |  |  |  |  |  |  |  |  |  |  |  |  |  |  |  |  |  |  |  |  |  |  |  |
| **As AdHc** |  | -0.242 |  | 0.037 |  | — |  |  |  |  |  |  |  |  |  |  |  |  |  |  |  |  |  |  |  |  |  |  |  |  |  |  |  |  |  |  |  |  |  |  |  |  |  |  |  |  |  |  |  |
| **As PMc** |  | 0.020 |  | 0.005 |  | 0.084 |  | — |  |  |  |  |  |  |  |  |  |  |  |  |  |  |  |  |  |  |  |  |  |  |  |  |  |  |  |  |  |  |  |  |  |  |  |  |  |  |  |  |  |
| **As SMc** |  | -0.202 |  | 0.071 |  | 0.216 |  | 0.072 |  | — |  |  |  |  |  |  |  |  |  |  |  |  |  |  |  |  |  |  |  |  |  |  |  |  |  |  |  |  |  |  |  |  |  |  |  |  |  |  |  |
| **AdEw** |  | 0.337 | ** | 0.132 |  | -0.311 | * | 0.068 |  | -0.089 |  | — |  |  |  |  |  |  |  |  |  |  |  |  |  |  |  |  |  |  |  |  |  |  |  |  |  |  |  |  |  |  |  |  |  |  |  |  |  |
| **AdEl** |  | -0.021 |  | 0.130 |  | -0.232 |  | 0.004 |  | -0.073 |  | 0.902 | *** | — |  |  |  |  |  |  |  |  |  |  |  |  |  |  |  |  |  |  |  |  |  |  |  |  |  |  |  |  |  |  |  |  |  |  |  |
| **AdHw** |  | -0.286 | * | 0.088 |  | 0.890 | *** | 0.053 |  | 0.109 |  | -0.167 |  | -0.064 |  | — |  |  |  |  |  |  |  |  |  |  |  |  |  |  |  |  |  |  |  |  |  |  |  |  |  |  |  |  |  |  |  |  |  |
| **AdHl** |  | -0.297 | * | 0.104 |  | 0.861 | *** | 0.070 |  | 0.089 |  | -0.157 |  | -0.055 |  | 0.994 | *** | — |  |  |  |  |  |  |  |  |  |  |  |  |  |  |  |  |  |  |  |  |  |  |  |  |  |  |  |  |  |  |  |
| **PMw** |  | -0.059 |  | 0.086 |  | 0.087 |  | 0.625 | *** | 0.006 |  | 0.313 | * | 0.294 | * | 0.180 |  | 0.196 |  | — |  |  |  |  |  |  |  |  |  |  |  |  |  |  |  |  |  |  |  |  |  |  |  |  |  |  |  |  |  |
| **PMl** |  | -0.045 |  | 0.058 |  | -0.035 |  | -0.600 | *** | -0.103 |  | 0.227 |  | 0.262 | * | 0.139 |  | 0.125 |  | 0.155 |  | — |  |  |  |  |  |  |  |  |  |  |  |  |  |  |  |  |  |  |  |  |  |  |  |  |  |  |  |
| **SMw** |  | -0.174 |  | 0.128 |  | 0.130 |  | 0.330 | ** | 0.095 |  | 0.254 | * | 0.264 | * | 0.219 |  | 0.217 |  | 0.768 | *** | 0.336 | ** | — |  |  |  |  |  |  |  |  |  |  |  |  |  |  |  |  |  |  |  |  |  |  |  |  |  |
| **SMl** |  | -0.075 |  | 0.178 |  | 0.058 |  | 0.316 | * | -0.148 |  | 0.302 | * | 0.278 | * | 0.154 |  | 0.156 |  | 0.758 | *** | 0.353 | ** | 0.951 | *** | — |  |  |  |  |  |  |  |  |  |  |  |  |  |  |  |  |  |  |  |  |  |  |  |
| **AbEw** |  | 0.059 |  | 0.271 | * | -0.203 |  | 0.111 |  | -0.115 |  | 0.572 | *** | 0.537 | *** | -0.024 |  | -0.014 |  | 0.502 | *** | 0.331 | ** | 0.565 | *** | 0.622 | *** | — |  |  |  |  |  |  |  |  |  |  |  |  |  |  |  |  |  |  |  |  |  |
| **AbEl** |  | 0.154 |  | -0.043 |  | -0.222 |  | 0.113 |  | -0.137 |  | 0.577 | *** | 0.513 | *** | -0.066 |  | -0.061 |  | 0.495 | *** | 0.328 | ** | 0.556 | *** | 0.610 | *** | 0.932 | *** | — |  |  |  |  |  |  |  |  |  |  |  |  |  |  |  |  |  |  |  |
| **LMA** |  | -0.166 |  | 0.026 |  | 0.409 | *** | 0.177 |  | 0.079 |  | -0.136 |  | -0.053 |  | 0.385 | ** | 0.369 | ** | 0.333 | ** | 0.148 |  | 0.384 | ** | 0.347 | ** | -0.107 |  | -0.109 |  | — |  |  |  |  |  |  |  |  |  |  |  |  |  |  |  |  |  |
| **LDMC** |  | -0.052 |  | -0.091 |  | -0.117 |  | -0.128 |  | 0.038 |  | -0.121 |  | -0.075 |  | -0.143 |  | -0.141 |  | -0.181 |  | -0.007 |  | -0.122 |  | -0.160 |  | -0.207 |  | -0.197 |  | 0.399 | ** | — |  |  |  |  |  |  |  |  |  |  |  |  |  |  |  |
| **CN** |  | -0.127 |  | 0.031 |  | 0.240 |  | 0.235 |  | 0.081 |  | 0.075 |  | 0.117 |  | 0.301 | * | 0.299 | * | 0.377 | ** | 0.158 |  | 0.372 | ** | 0.350 | ** | -0.038 |  | -0.046 |  | 0.803 | *** | 0.371 | ** | — |  |  |  |  |  |  |  |  |  |  |  |  |  |
| **C area** |  | -0.193 |  | 0.064 |  | 0.388 | ** | 0.195 |  | 0.051 |  | -0.133 |  | -0.049 |  | 0.365 | ** | 0.354 | ** | 0.361 | ** | 0.143 |  | 0.399 | ** | 0.375 | ** | -0.050 |  | -0.063 |  | 0.982 | *** | 0.408 | ** | 0.799 | *** | — |  |  |  |  |  |  |  |  |  |  |  |
| **N area** |  | -0.136 |  | -0.040 |  | 0.255 | * | -0.034 |  | 0.029 |  | -0.317 | * | -0.232 |  | 0.079 |  | 0.058 |  | -0.075 |  | -0.115 |  | -0.050 |  | -0.086 |  | -0.113 |  | -0.103 |  | 0.228 |  | 0.012 |  | -0.312 | * | 0.253 | * | — |  |  |  |  |  |  |  |  |  |
| **A max area** |  | -0.033 |  | 0.004 |  | 0.102 |  | -0.212 |  | -0.132 |  | -0.242 |  | -0.213 |  | 0.027 |  | 0.019 |  | -0.234 |  | 0.005 |  | -0.286 | * | -0.260 | * | -0.169 |  | -0.181 |  | -0.070 |  | -0.021 |  | -0.167 |  | -0.080 |  | 0.152 |  | — |  |  |  |  |  |  |  |
| **C mass** |  | -0.239 |  | 0.180 |  | 0.168 |  | 0.250 | * | -0.032 |  | -0.045 |  | -0.014 |  | 0.164 |  | 0.184 |  | 0.340 | ** | -0.030 |  | 0.319 | * | 0.349 | ** | 0.157 |  | 0.116 |  | 0.504 | *** | 0.260 | * | 0.481 | *** | 0.636 | *** | 0.245 |  | -0.116 |  | — |  |  |  |  |  |
| **N mass** |  | 0.059 |  | 0.018 |  | -0.194 |  | -0.202 |  | -0.099 |  | -0.133 |  | -0.153 |  | -0.265 | * | -0.260 | * | -0.332 | ** | -0.169 |  | -0.325 | ** | -0.297 | * | 0.055 |  | 0.046 |  | -0.726 | *** | -0.355 | ** | -0.971 | *** | -0.691 | *** | 0.440 | *** | 0.161 |  | -0.299 | * | — |  |  |  |
| **A max mass** |  | 0.108 |  | -0.003 |  | -0.250 | * | -0.314 | * | -0.177 |  | -0.096 |  | -0.136 |  | -0.294 | * | -0.286 | * | -0.384 | ** | -0.042 |  | -0.442 | *** | -0.380 | ** | -0.011 |  | -0.013 |  | -0.744 | *** | -0.312 | * | -0.705 | *** | -0.742 | *** | -0.048 |  | 0.666 | *** | -0.470 | *** | 0.645 | *** | — |  |

**Fig. S1.** Principal component analysis (PCA) including foliar and anatomical traits showing correlations between each variable and the scores of the first axis (a) and second axis of the PCA (b). Acronyms are as follows: %Adaxial cuticle thickness (AdCT, %), %Adaxial epidermis thickness (AdET, %), %Palisade mesophyll thickness (PMT, %), %Spongy mesophyll thickness (SMT, %), Air space (AS, µm^2^), Vascular bundle diameter (VBD, µm), Leaf thickness (LT, µm), Pallisate/Spongy thickness ratio (PMT/SMT), A _max mass_ (A max, nmol g^-1^ s^-1^), Leaf Mass per Area (LMA, g m^-2^), Leaf Nitrogen Content (LNC _mass_, mg g^-1^), Leaf Carbon Content (LCC _mass_, mg g^-1^), CN ratio (Carbon/Nitrogen ratio), and Leaf Dry Matter Content (LDMC, mg g^-1^).
